# GbSER02 Interacts With GhVOZ1 to Promote Fiber Elongation by Modulating Gibberellin Synthesis in Cotton

**DOI:** 10.1002/advs.202417578

**Published:** 2025-06-25

**Authors:** Hao Jia, Dongmei Zhang, Qishen Gu, Yanbin Li, Jing Li, Wannian Li, Zhengwen Sun, Huifeng Ke, Jun Yang, Liqiang Wu, Yan Zhang, Zhiying Ma, Xingfen Wang

**Affiliations:** ^1^ North China Key Laboratory for Crop Germplasm Resources of Education Ministry Key Laboratory for Crop Germplasm Resources of Hebei State Key Laboratory of North China Crop Improvement and Regulation Collaborative Innovation Center of Cotton Industry in Hebei Hebei Agricultural University Baoding 071001 China

**Keywords:** Cotton, fiber length, GbSER02, GhGA3ox1, GhVOZ1, SNP

## Abstract

The elongation period of cotton fiber development is crucial for ensuring cotton quality. However, the molecular mechanisms underlying fiber elongation remain unclear. This study reveals that *SER02*_*A11*, which is preferentially expressed during the fiber elongation period, encodes serine protease inhibitors (serpins). A previously uncharacterized SNP^517th^ is localized in the gene's coding region between *Gossypium barbadense* and *G. hirsutum*, leading to the premature translation termination and low expression of *GhSER02* in *G. hirsutum*. However, in *G. barbadense*, normal protein translation and high expression of *GbSER02* are observed. Ectopic *GbSER02* expression or site‐directed *GhSER02'* mutagenesis (but not *GhSER02*) promotes trichome and root cell elongation in *Arabidopsis*, indicating that SNP^517th^ causes the dysfunction of *GhSER02*. Overexpressing *GbSER02* in *G. hirsutum* increases fiber length. GbSER02 interacts with the transcription factor GhVOZ1 (vascular one‐zinc‐finger protein) in the cytoplasm, alleviating the inhibitory effect of GhVOZ1 on *GhGA3ox1* expression, thereby promoting gibberellin synthesis. Subsequently, the cell wall loosening‐related genes *GhXTH2* and *GhEXPA1* are significantly upregulated whereas the flavonoid metabolism‐related gene *GhCHS1* is significantly downregulated, ultimately improving fiber length. Collectively, this study reveals the essential role of the GbSER02‐GhVOZ1‐GhGA3ox1 module in regulating fiber quality and provides novel insights into the mechanism of fiber development in cotton.

## Graphical abstract

*GbSER02* with a single base change (SNP^517G^) encodes normal serpin protein and interacts with transcription factor VOZ1 to alleviate the repression of VOZ1 on *GA3ox1* and promote GA_3_ biosynthesis. Further, GA_3_ induces the expression of cell wall loosening‐related genes and decreases flavonoid content, ultimately facilitating fiber cell elongation in cotton.

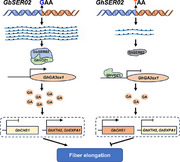

## 1 Introduction

Cotton is a globally significant cash crop and a major natural textile. Cotton fibers are unicellular structures that arise from the trichome primordia in the epidermis of the ovule.^[^ ^1^, ^2^ ^]^ Fiber growth and morphogenesis undergo four key developmental stages: initiation, elongation, secondary cell wall formation, and maturation.^[^ ^3^ ^]^ Fiber elongation has gained increasing attention owing to its critical role in determining fiber yield and quality. Various plant hormones play significant roles in cotton fiber elongation.^[^ ^4^ ^]^ For instance, gibberellin (GA) is an extensively studied hormone involved in cotton fiber development. Endogenous GA levels are significantly upregulated by the overexpression of *GhGA20ox1*, which encodes GA 20‐oxidase1, resulting in the formation of more and longer fibers on the ovule surface in cotton.^[^ ^5^ ^]^ However, the GA signaling pathway is significantly negatively regulated by DELLA proteins. Mechanistically, the cotton DELLA protein “Slender rice 1” (GhSLR1) specifically interacts with the transcription factor homeodomain protein 3 (*GhHOX3*), inhibiting the expression of downstream genes associated with fiber elongation. When GA levels are elevated, GhSLR1 is degraded, releasing *GhHOX3* and activating the expression of target genes to promote fiber elongation.^[^ ^6^ ^]^ GA acts upstream of strigolactones (SLs), regulates their biosynthesis, and promotes the accumulation of very‐long‐chain fatty acids in fibers, thereby facilitating cell elongation.^[^ ^7^ ^]^ GA degrades the DELLA protein SLR1, thereby alleviating its inhibitory effects on *GhZFP8* and *GhBLH1*. The resulting free *GhZFP8* and *GhBLH1* subsequently bind to the promoters of *GhSDCP1* and *GhKCS12*, respectively, thereby activating their expression. GA promotes fiber elongation through these two parallel signaling cascades.^[^ ^8^ ^]^ Although significant progress has been made in elucidating the functions of GA in cotton fiber cell elongation, studies on the upstream regulatory mechanisms of GA are relatively scarce.

In our previous study, multiple differentially expressed genes were identified across various fiber developmental stages between *G. barbadense* and *G. hirsutum*.^[^ ^9^ ^]^ Among these, a gene encoding a serine protease inhibitor (serpin) showed predominant expression during the fiber elongation period in *G. barbadense* and *G. hirsutum*, although its expression was higher in *G. barbadense*. Serpins, the largest family of protease inhibitors, are widely distributed across various organisms, including bacteria, fungi, plants, and humans.^[^ ^10^ ^]^ While dozens of intra‐ and extracellular animal serpins have been functionally characterized, relatively little is known about their functions in plants.^[^ ^11^, ^12^ ^]^ Generally, serpin proteins are structurally well‐conserved. A typical serpin molecule is composed of three β‐sheets (A–C), eight to nine α‐helices (A–H), and a reactive central loop (RCL) essential for the function of inhibitory serpins.^[^ ^13^ ^]^ The RCL acts as a substrate for the target protease, and when they bind together, serpin and protease form a Michaelis encounter complex.^[^ ^14^, ^15^ ^]^ Following the formation of this complex, the protease cleaves at the peptide bond linking the P1 and P1′ residues in the reactive center.^[^ ^16^ ^]^ The most common plant serpin reactive center is P2‐P1‐P1′ (Leu‐Arg‐Xaa), wherein Xaa represents a small residue (Ala, Cys, Gly or Ser), such as BSZx^[^ ^17^ ^]^ or AtSerpin1,^[^ ^18^ ^]^ which are known as “LR serpins.”^[^ ^12^ ^]^ AtSerpin1, a paradigmatic plant “LR serpin,” interacts with protease RD21.^[^ ^18^ ^]^ Together with other “LR serpins” from *Oryza sativa* ^[^ ^19^ ^]^ and *Medicago truncatula*,^[^ ^20^ ^]^ they were found to be negative regulators of cell death. Excluding the LR serpins, the arrangement of most plant serpin clades in a species‐specific manner suggests that extant plant serpins evolved during plant speciation.^[^ ^21^ ^]^ Analysis of serpin sequences from wheat grains showed that the majority contained the reactive center P1‐P1′ (Gln‐Gln or X‐Gln), whereas reactive centers in most rye grain serpins contained P2‐P1‐P1′ (Gln‐Gln‐Ser or X‐Gln‐Ser).^[^ ^22^, ^23^ ^]^ The serpins present in seeds may be associated with different functions, such as energy storage and defense.^[^ ^22^ ^]^ However, whether serpins are involved in cotton fiber elongation remain unknown.

This study revealed that a single nucleotide polymorphism (SNP) mutation (G to T) in the coding region of the serpin gene *GhSER02* causes the premature termination of translation and loss of the RCL domain. However, its orthologous gene *GbSER02* from *G. barbadense* is complete. Furthermore, a comparison of the functional differences between *GhSER02* and *GbSER02* in *Arabidopsis* was performed, and the function and mechanism of *GbSER02* in fiber development were elucidated using a *GbSER02*‐overexpressing line of cotton. A Kompetitive Allele‐Specific PCR (KASP) marker was developed and validated. These findings provide new insights into the molecular mechanisms underlying fiber development and may facilitate molecular breeding programs to improve cotton fiber quality.

## 2 Results

### 2.1 *GbSER02* is Preferentially Expressed in Cotton Fiber and Possesses an Elite SNP Site

**Figure 1:**
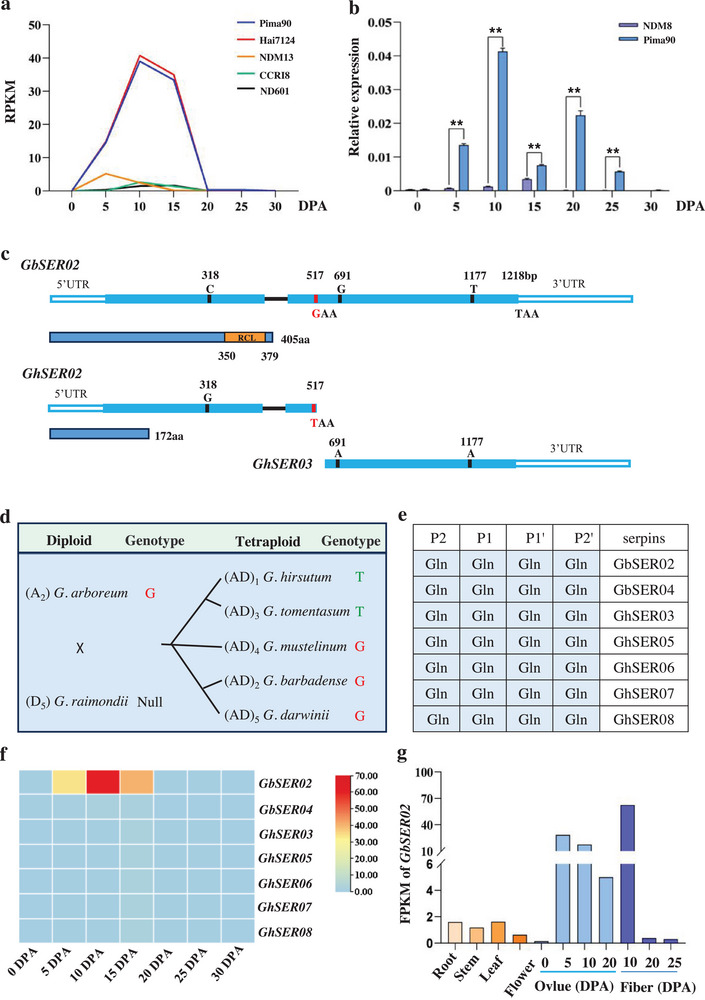
*GbSER02*, which is preferentially expressed in cotton fibers, possesses an elite SNP site. a) Expression of *SER_A11* at different time points of fiber development between *G. barbadense* and *G. hirsutum* based on RNA‐Seq data. b) qRT‐PCR analysis of *SER_A11* at different time‐points of fiber development in *G. barbadense* Pima90 and *G. hirsutum* NDM8. ** Significant difference at *P* < 0.01 by Student's *t*‐test. c) Schematic diagrams of nucleotide and amino acid sequences of *GbSER02*, *GhSER02* and *GhSER03*. SNPs are presented at positions 318^th^, 517^th^, 691^th^, and 1177^th^ among *GbSER02*, *GhSER02*, and *GhSER03*. An SNP (G/T) variation at position 517^th^ leads to the premature termination of the *SER* gene in *G. hirsutum* and formation of two genes (*GhSER02* and *GhSER03*). RCL represents reactive central loop. d) Evolutionary analysis of *SER_A11* in cotton species. e) Key amino acid residues in the RCL sequences of GbSERs and GhSERs. f) Expression heatmap of rich Q (RQ)‐type *Serpins* from *G. barbadense* and *G. hirsutum* at different time points of fiber development. g) Expression of *GbSER02* in various tissues based on RNA‐seq data.

RNA‐seq data from different fiber development stages of *G. barbadense* and *G. hirsutum* revealed that a serpin gene located on chromosome A11 displayed obviously differential expression during the fiber elongation stage between the two species (**Figure** **1**a). Furthermore, the qRT‐PCR assay validated the significantly high expression of the serpin gene in the fibers at 10 days post‐anthesis (DPA) in *G. barbadense* (Figure 1b). We cloned two open reading frame (ORF) sequences from *G. barbadense* and *G. hirsutum* based on the coding sequence (CDS) of *GbM_A11G0710* (named *GbSER02*). The ORF of *GbSER02* was 1218 bp long and encoded 405 amino acids (aa), whereas the gene sequence cloned from *G. hirsutum* contained an SNP (G/T) at position 517^th^, thus forming a stop codon (TAA) and resulting in a truncated protein (GhSER02, 172 aa) without the RCL domain. Furthermore, a new start codon (ATG) appeared in the subsequent sequence, forming another truncated gene (*GhSER03*; Figure 1c). Considering the absence of a ribosome‐scanning sequence in *GhSER03*, we speculated that it is likely a pseudogene. To verify the reliability of SNP^517th^, we cloned the *Serpin_A11* gene from several cotton varieties from *G. barbadense* and *G. hirsutum* and aligned the sequences with publicly available genome data. The results showed that all serpin genes in the diploid *G. arboreum* and most tetraploid species (*G. mustelinum*, *G. darwinii*, and *G. barbadense*) exhibited a guanine (G‐type), whereas the genes in *G. hirsutum* and *G. tomentasum* consistently exhibited a thymine (T‐type; Figure S1, Supporting Information). Evolutionary analysis suggested that the different sites arose during the divergence of *G. barbadense* and *G. hirsutum* (Figure 1d).

To comprehensively elucidate the serpin genes (*SER*) in cotton, we analyzed all *SERs* across the *G. barbadense* and *G. hirsutum* genomes and identified four *SERs* from the *G. barbadense* genome and eight *SERs* from the *G. hirsutum* genome. Notably, two adjacent *GhSER* genes were located on chromosome A11 in *G. hirsutum* whereas only one *SER* gene was found in *G. barbadense*. We designated the serpin gene family members according to their chromosomal locations (Figure S2, Supporting Information). Several reports have indicated that the sequence P2‐P1‐P1′‐P2′ likely contains the most critical residues for determining inhibitory specificity in most serpins.^[^ ^15^, ^24^, ^25^, ^26^ ^]^ “LR serpins” are widely distributed throughout the plant kingdom.^[^ ^12^ ^]^ In cotton, two GbSERs (GbSER02, GbSER04) and five GhSERs (GhSER03, GhSER05, GhSER06, GhSER07, GhSER08) contain the P2‐P1‐P1′‐P2′ (Gln‐Gln‐Gln‐Gln) sequence and belongs to the rich Q (RQ) type of serpins, which are different from LR serpins (Leu‐Arg‐Xaa; Figure 1e). This may be attributed to RCL hypervariability, which facilitates the rapid evolution of new functions in specific tissues in response to environmental conditions. Using our transcriptome data from the fiber development of *G. barbadense* and *G. hirsutum*, only *GbSER02* of the seven cotton *Serpins* was determined to be highly expressed in the 10 DPA fibers (Figure 1f). We further examined the tissue specificity of *GbSER02* using publicly available data from the cotton database (http://cotton.zju.edu.cn) and found that *GbSER02* is expressed at the highest level in 10 DPA fibers (Figure 1g). Thus, *GbSER02* may participate in fiber development processes, especially fiber elongation.

### 2.2 G/T SNP^517th^ of *GbSER02* and *GhSER02* is a Crucial Variation in Regulating Cell Length

**Figure 2:**
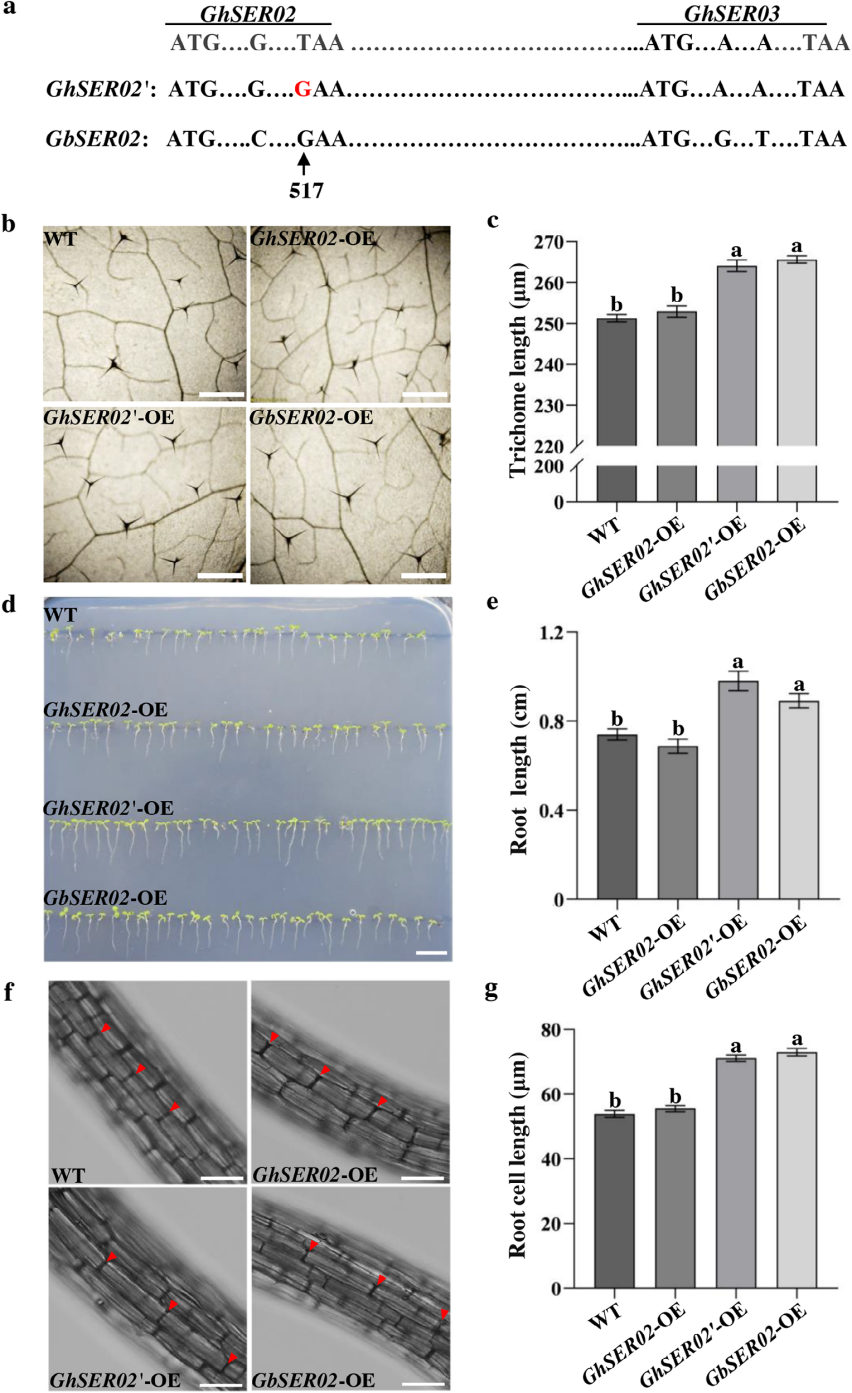
SNP^517th^ in *SER_A11* is a crucial variation between *GbSER02* and *GhSER02* for regulating the cell length of *Arabidopsis*. a) Comparison of multiple SNP sites among *GbSER02*, *GhSER02′*, and *GhSER02*. The 517^th^ T of *GhSER02* was substituted with G, resulting in the new sequence *GhSER02′*. b) Leaf trichome observations of the WT and overexpression lines (*GhSER02*‐OE, *GhSER02′*‐OE, and *GbSER02*‐OE) under a 250× optical microscope. Scale bars, 500 µm. c) Trichome length statistics of the WT and transgenic lines. d) Image of the root length of the WT and overexpressing lines. Scale bar, 20 mm. e) Root length statistics of the WT and overexpressing lines. f) Root cell length observations and g) Statistical analysis of the WT and overexpressing lines. Scale bars, 50 µm. Data are presented as means ± SE. Lowercase letters represent significant differences according to Tukey's HSD test.

As a serpin, *GhSER02* lacks the RCL, which is crucial for its inhibitory function, this potentially leads to functional changes. To determine the effect of this specific mutation, we performed site‐directed mutagenesis (T^517th^ to G^517th^) in the *GhSER02* sequence and generated a new sequence designated as *GhSER02′* (**Figure** **2**a). *Arabidopsis* leaf trichomes provide a useful model for studying cotton fiber development as they share certain regulatory mechanisms.^[^ ^27^, ^28^, ^29^ ^]^ We overexpressed *GhSER02*, *GhSER02′*, and *GbSER02* in *Arabidopsis thaliana*, selected transgenic lines based on herbicide resistance, and generated homozygous lines by self‐pollination over two generations. When the seedlings developed 5–6 rosette leaves, the fifth true leaf was harvested to measure the trichome length. The results indicated that both the *GhSER02′*‐OE (264.07 ± 29.19 µm) and *GbSER02*‐OE (265.60 ± 24.42 µm) lines exhibited significantly longer trichomes compared to those of *GhSER02*‐OE (252.91 ± 26.26 µm) or wild type (WT; 251.26 ± 25.61 µm). However, significant differences were not observed between the *GhSER02′*‐OE and *GbSER02*‐OE lines or *GhSER02*‐OE line and WT (Figure 2b,c). Additionally, overexpressing *GhSER02′* and *GbSER02* promoted root elongation in *Arabidopsis*, with more pronounced phenotypic effects in the *GhSER02*'‐OE (0.98 ± 0.13 cm) and *GbSER02*‐OE (0.89 ± 0.15 cm) lines (Figure 2d,e). The root cell length of the *GhSER02′*‐OE (71.10 ± 9.08 µm) and *GbSER02*‐OE (72.98 ± 8.58 µm) lines were significantly longer than those of the WT (53.87 ± 8.08 µm) and *GhSER02*‐OE (55.55 ± 7.68 µm; Figure 2f,g). This suggests that *GhSER02′* and *GbSER02* promote trichome and root elongation in *Arabidopsis*, whereas *GhSER02* is a dysfunction gene.

### 2.3 *GbSER02* Positively Regulates Fiber Length

**Figure 3:**
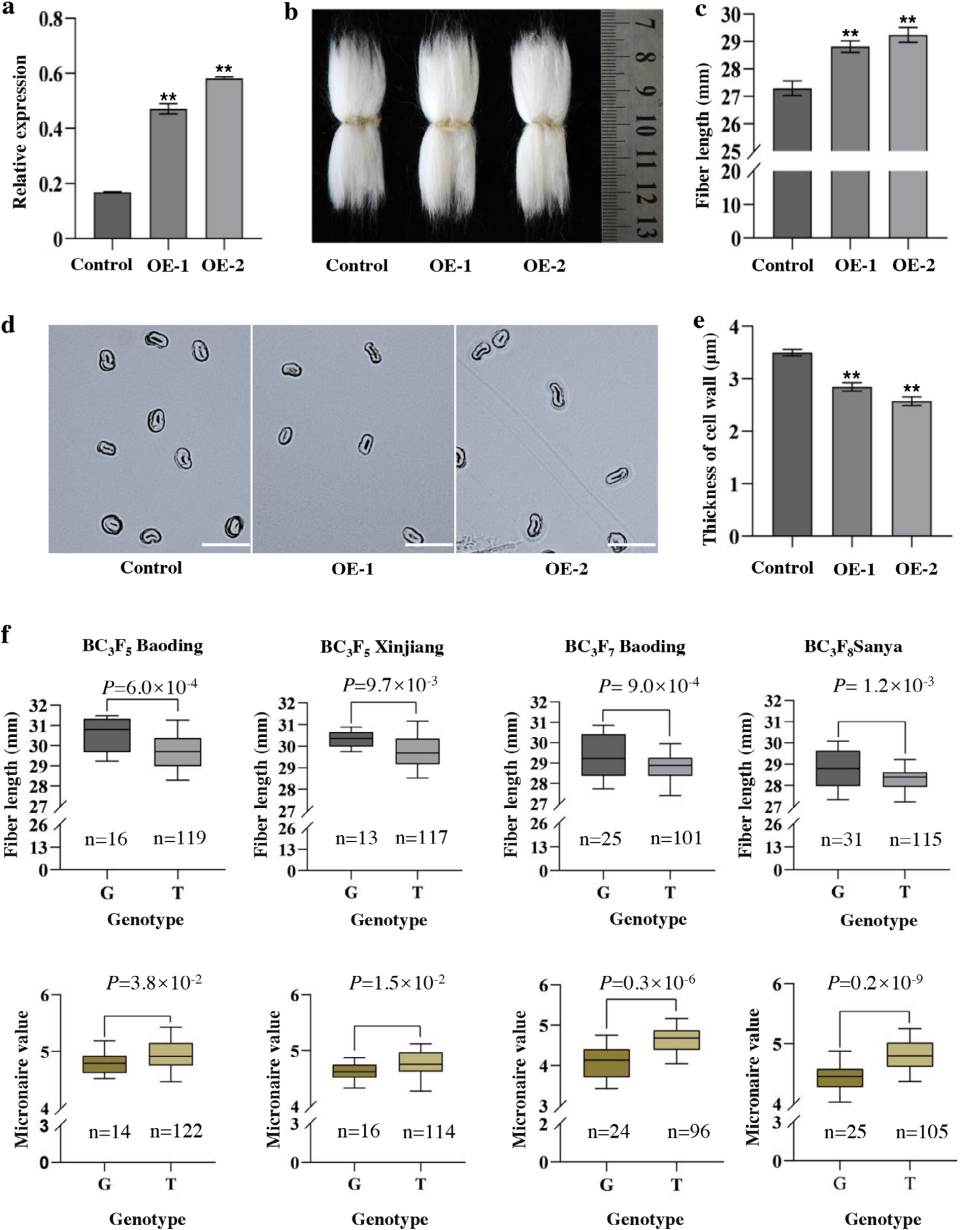
*GbSER02* positively regulated fiber length in cotton. a) Expression of *GbSER02* in the control and overexpressing cotton lines (OE‐1, OE‐2). b) Representative fiber length images of the control, OE‐1, and OE‐2. c) Fiber length measurement of the control and *GbSER02*‐OE lines. d) Cross sections of mature fibers from the control and *GbSER02*‐OE lines. Scale bars, 20 µm. e) Comparison of the fiber cell wall thickness between the control and *GbSER02*‐OE lines. f) Statistical analysis of fiber length and Micronaire value in the CSSL lines. C and G represent the *SER02* ^*CC*^ and *SER02^GG^* lines in the CSSL population, respectively. (Data were collected from BC_3_F_5_, Baoding and Xinjiang in 2016; BC_3_F_7_, Baoding in 2022; BC_3_F_8_, Sanya in 2022). Statistical significance was determined using two‐tailed Student's *t*‐test.

To further investigate the role of *GbSER02* in fiber development, we created overexpression lines in *G. hirsutum* using the ubiquitin (Ubi) promoter. The results of cross‐vector primer amplification and product recovery sequencing confirmed that *GbSER02* was successfully transformed into the receptor cotton Jin668 (Figure S3, Supporting Information). qRT‐PCR analysis revealed that *GbSER02* transcript levels in the fibers of the two overexpression lines were 1.5–2.5‐fold higher than those in the non‐transgenic control (**Figure** **3**a). The *GbSER02*‐OE line produced longer fibers than the control (Figure 3b). The average length of mature fibers in the OE‐1 line (28.81 ± 0.17 mm) and OE‐2 line (29.24 ± 0.06 mm) was 5.6% and 7.1% longer, respectively, than that of the control (27.29 ± 0.22 mm; Figure 3c). The thickness of the mature fiber cell wall was observed using a microscope, and the cross sections of the transgenic cotton fibers displayed a significant reduction in the thickness of the secondary cell wall (Figure 3d). The thickness of the control fiber was 3.50 ± 0.40 µm, whereas the transgenic fibers were 2.84 ± 0.44 and 2.57 ± 0.43 µm for lines OE‐1 and OE‐2, respectively (Figure 3e). Moreover, fiber quality measurements revealed that the Micronaire value of the fibers was significantly lower in the OE lines than in the control, whereas the fiber strength did not significantly differ between them (Figure S4, Supporting Information). These results demonstrate that *GbSER02* positively regulates fiber length and fineness.

To further employ the gene in the molecular breeding of fiber quality, we developed a KASP marker based on SNP^318th^, which is closely linked to SNP^517th^ (Table S1 and Figure S5, Supporting Information). This marker was validated using the chromosomal segment substitution line (CSSL) population with *G. barbadense* segments (Figure S6, Supporting Information). Several C‐type lines were identified with significantly longer fibers and lower Micronaire values than the G‐type lines based on the fiber quality data collected from the four environments (Figure 3f), indicating that the KASP marker was effective.

### 2.4 GbSER02 Interacts with Transcriptional Factor GhVOZ1

**Figure 4:**
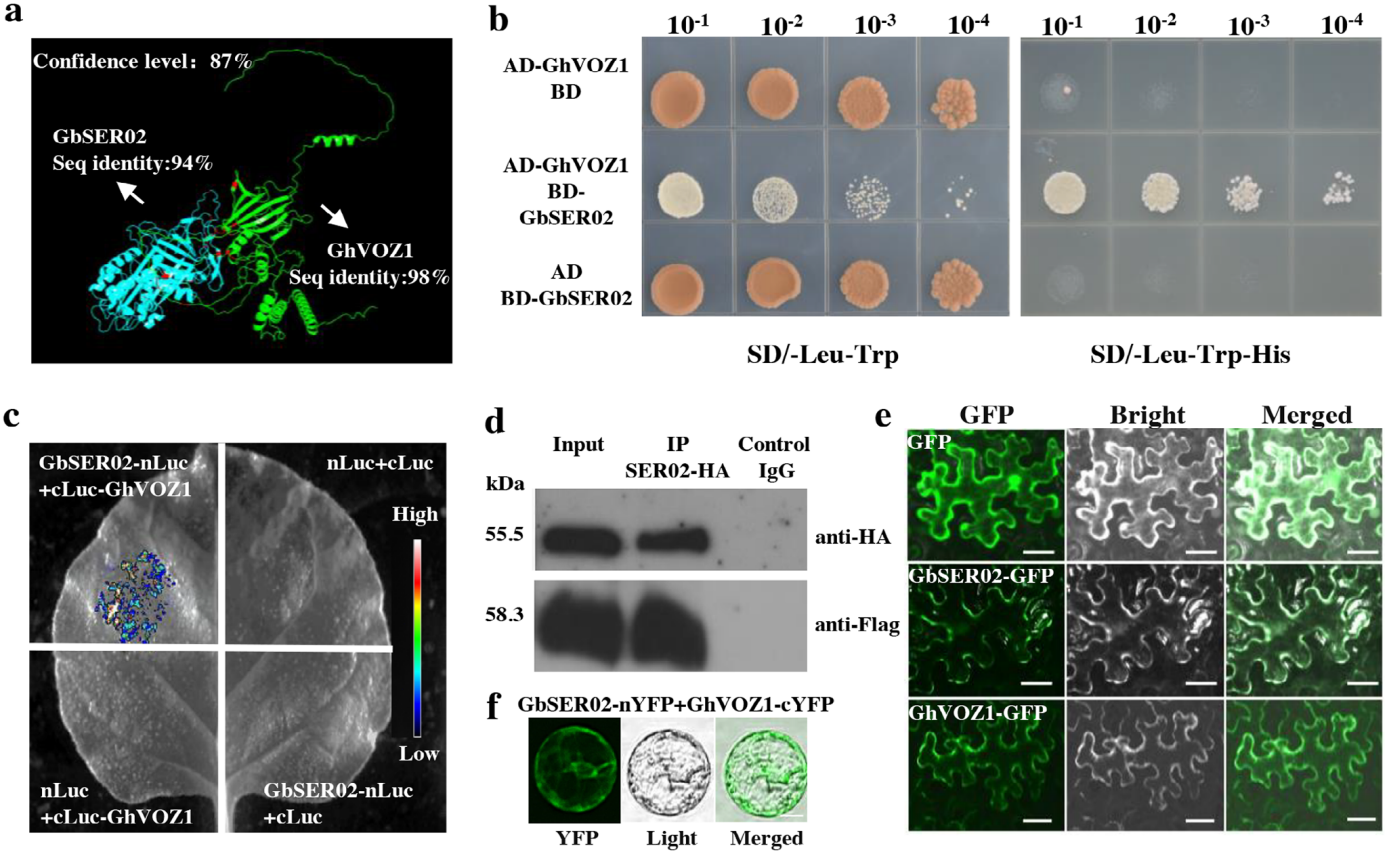
GbSER02 interacted with transcription factor GhVOZ1. a) Interaction between GbSER02 and GhVOZ1 were predicted by AlphaFold with 87% confidence (<50%, non‐interaction; >50% and <80%, medium interaction; >80%, high interaction). b) Interaction validation of GbSER02 and GhVOZ1 using the yeast two‐hybrid system. c) Interaction validation of GbSER02 and GhVOZ1 using luciferase complementation imaging (LCI) assay. d) Co‐IP assay validated the interaction between GbSER02 and GhVOZ1 in *N. benthamiana*. e) Subcellular localization of GbSER02 and GhVOZ1 in *N. benthamiana* epidermal cells. Scale bars, 50 µm. f) BiFC assay validated the interaction of GbSER02 and *GhVOZ1* in cotton protoplasts. Scale bar, 20 µm.

To investigate the molecular mechanism that *GbSER02* influences cotton fiber quality, we screened *GhVOZ1* (*GhM_A03G2071*) from a yeast library of fiber tissues. Using AlphaFold, we predicted an 87% confidence level for the interaction between GbSER02 and GhVOZ1 (**Figure** **4**a), suggesting the strong likelihood of an interaction. We experimentally confirmed this interaction using yeast two‐hybrid (Y2H), split‐luciferase (LUC), co‐immunoprecipitation (Co‐IP), and bimolecular fluorescence complementation (BiFC) assays. The Y2H results showed that yeast cells carrying GhVOZ1‐AD and GbSER02‐BD grew normally on SD‐Leu‐Trp‐His selective medium, whereas cells carrying GhVOZ1‐AD/BD or AD/GbSER02‐BD did not grow, indicating that GbSER02 interacted with GhVOZ1 (Figure 4b). The LUC assay demonstrated that the co‐infiltration of leaves with the GbSER02‐nLUC and cLUC‐GhVOZ1 constructs engendered strong luciferase activity, whereas no signal was observed in the negative control in *Nicotiana benthamiana* (Figure 4c). We further confirmed this interaction using a co‐IP assay in *N. benthamiana* co‐transfected with constructs expressing GbSER02‐HA and GhVOZ1‐Flag. With hyaluronic acid (HA)‐coupled agarose beads, GbSER02‐HA was pulled down and the GhVOZ1‐Flag protein was detected using anti‐flag antibodies (Figure 4d). To determine the subcellular location of their interactions, we performed subcellular localization and BiFC assays, and found that fluorescence signals appeared in the cytoplasm when GbSER02‐GFP or GhVOZ1‐GFP was transformed into *N. benthamiana* or when GbSER02‐nYFP and GhVOZ1‐cYFP were co‐transformed into cotton protoplasts, indicating that GbSER02 and GhVOZ1 interacted in the cytoplasm (Figure 4e,f).

### 2.5 GhVOZ1 Binds to the Promoter of GA3ox1 to Inhibit GA_3_ Biosynthesis

**Figure 5:**
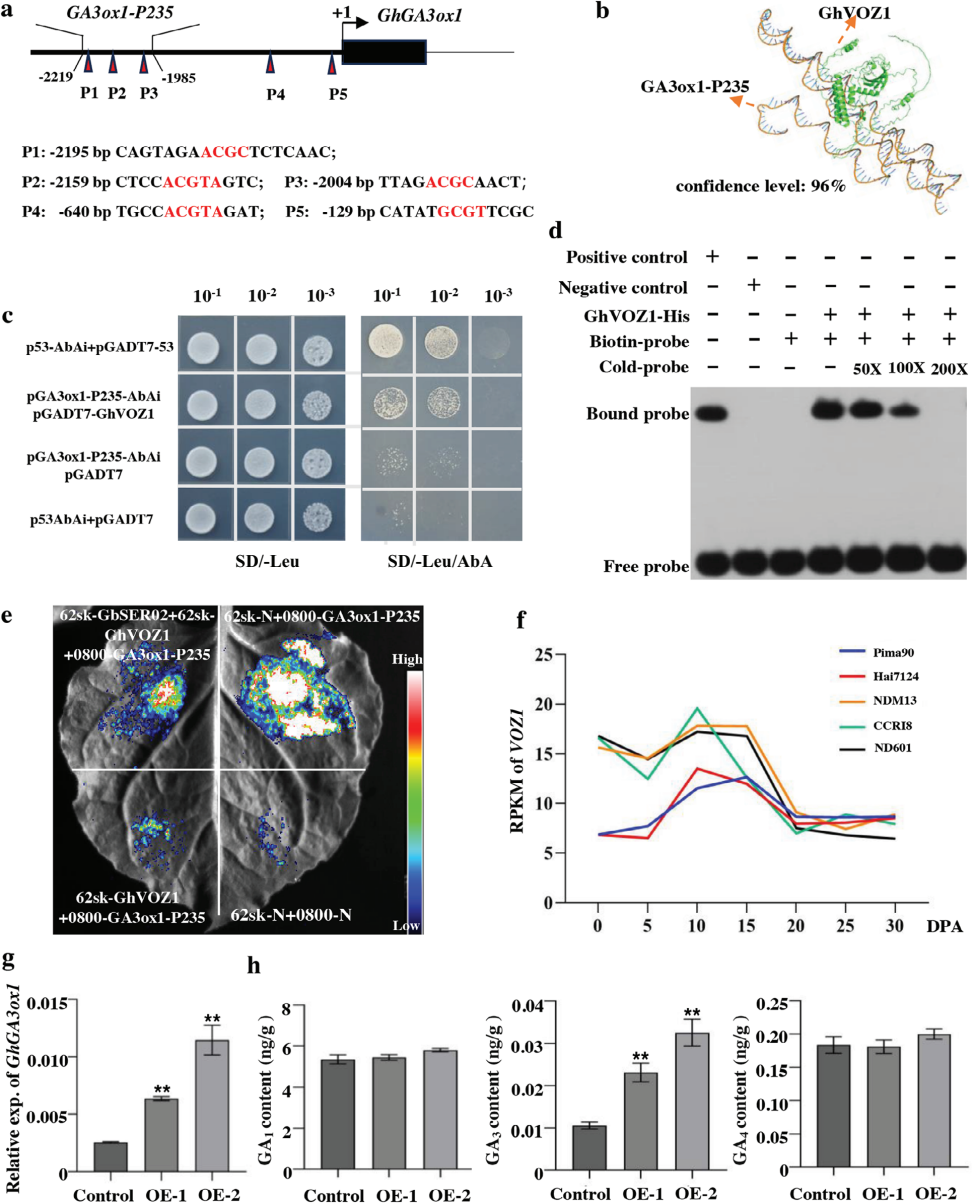
GhVOZ1 binds to the promoter of *GhGA3ox1* to repress GA_3_ biosynthesis. a) Binding site prediction of GhVOZ1 in the 2500 bp promoter upstream of *GhGA3ox1 (GhM_D10G0770)*. Binding motifs are enriched in the 235 bp region from −2219 to −1985 (*GA3ox1‐P235*). The red letters indicate the binding motifs. b) Prediction of the interaction between GhVOZ1 and *GA3ox1‐P235* based on the AlphaFold model. c) Yeast one hybrid results. d) EMSA assay showing that GhVOZ1 directly binds to the P1 motif of the *GhGA3ox1* promoter. e) Transactivation assay demonstrating the effect of GbSER02 and GhVOZ1 on *GhGA3ox1*. f) Expression of *VOZ1* at different time points of fiber development between *G. barbadense* and *G. hirsutum* based on RNA‐Seq data. g) Relative expression of *GhGA3ox1* in the control and OE cotton lines. h) Content of different GAs in the control and OE lines. ** Significant difference at *P* < 0.01 using Student's *t*‐test.

Previous studies showed that AtVOZ2 bound to the promoter of *Gibberellin 3β‐hydroxylase* (*GA3ox1*), a key gene in the GA biosynthesis pathway, and inhibited its expression.^[^ ^30^ ^]^ To reveal the regulatory mechanism of GhSER02‐GhVOZ1 in fiber quality, we silenced *GhVOZ1* in cotton and found that the expression of two GA biosynthesis genes, *GhKAO2* (*GhM_A08G2098*) and *GhGA3ox1* (*GhM_D10G0770*), were significantly upregulated in the silenced cotton, particularly *GhGA3ox1* (Figure S7, Supporting Information). This suggests that *GhVOZ1* may inhibit *GhGA3ox1* expression. Previous studies confirmed that both *AtVOZ2* and *SlVOZ1* bound to the motifs ACGC and ACGTA in the promoters of *AtGA3ox1* and *SlSFT*, respectively.^[^ ^30^, ^31^ ^]^ A comparison of the amino acid sequences of GhVOZ1, AtVOZ2, and SlVOZ1 showed that the domain‐B region in VOZ proteins was highly conserved (identity: 74.5%; Figure S8, Supporting Information), implying that GhVOZ1 might have similar binding sites for AtVOZ2 and SlVOZ1. We predicted five binding sites in the promoters (upstream 2.5 kb) of *GhM_D10G0770* (**Figure** **5**a) to select upstream sequences (−1985 to −2219, 235 bp) rich in binding motifs to predict the interaction between GhVOZ1 and *GA3ox1*‐*p235* and obtained a 96% confidence level (Figure 5b). Yeast one hybrid (Y1H) assay confirmed that *GhVOZ1* bound to the promoter of *GhGA3ox1* (Figure 5c). Electrophoretic mobility shift assay (EMSA) validated that GhVOZ1 directly bound to the selected GA3ox1 promoter probe (CAGTAGAACGCTCTCAAC) in vitro (Figure 5d). Furthermore, we used the abovementioned upstream sequence to promote the LUC reporter gene and determine whether GhVOZ1 regulated its transcription. Co‐infiltration of GhVOZ1 with the LUC reporter obviously reduced the luminescent signals compared to that of the control, demonstrating GhVOZ1's repressive effect on *GhGA3ox1* promoter activity. However, co‐infiltration of GbSER02 and GhVOZ1 with the LUC reporter enhanced the fluorescence intensity compared with that of GhVOZ1 alone (Figure 5e). According to transcriptome data from different fiber developmental stages, in *G. barbadense, VOZ1* expression was observed to be significantly lower than that in *G. hirsutum* during fiber elongation, indicating that GbSER02 alleviated GhVOZ1's inhibition of *GhGA3ox1* (Figure 5f).

To validate whether the GA content was affected by GbSER02‐GhVOZ1‐GhGA3ox1, the expression of *GhGA3ox1* and content of three active GAs (GA_1_, GA_3_, and GA_4_) were estimated in 10 DPA fibers of GbSER02‐OE‐1 and OE‐2. Therefore, *GhGA3ox1* expression was significantly upregulated and the GA_3_ content was significantly increased, however, the other GAs did not differ from the control (Figure 5g,h). Thus, *GbSER02* regulated GA_3_ levels in fibers via *GhVOZ1*.

### 2.6 GbSER02 Regulates Cell Wall Loosening and Flavonoid Metabolism‐Related Genes by GA

**Figure 6:**
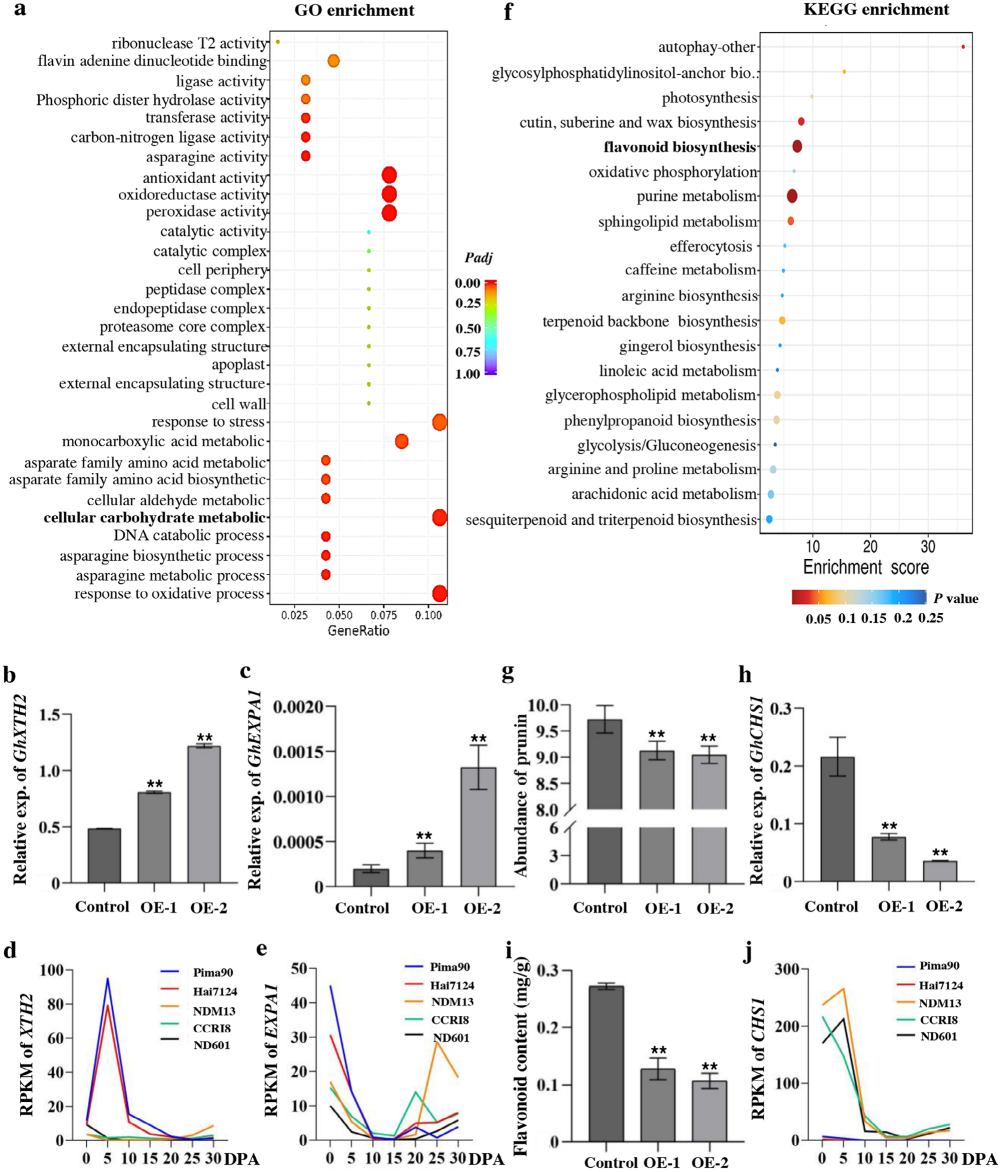
*GbSER02* regulates genes related to cell wall loosening and flavonoid metabolism by GA. a) Gene Ontology (GO) enrichment analysis of upregulated genes from 10 DPA fibers of *GbSER02*‐OE cotton lines. b) Relative expression of *GhXTH2* in the control and OE cotton lines. c) Relative expression of *GhEXPA1* in the control and OE cotton lines. d) Expression of *XTH2* at different time points of fiber development between *G. barbadense* and *G. hirsutum* based on the RNA‐Seq data. e) Expression of *EXPA1* at different time points of fiber development between *G. barbadense* and *G. hirsutum* based on RNA‐Seq data. f) KEGG enrichment analysis of the metabolites from fibers in the control and OE cotton lines. g) Abundance of flavonoid‐related prunin. h) Expression level of *GhCHS1* in the control and OE fibers. i) Flavonoid content in the control and OE lines. j) *CHS1* expression at different time points of fiber development between *G. barbadense* and *G. hirsutum* based on RNA‐Seq data. Data represent mean ± SD (Student's *t*‐test, **P < 0.01).

To further elucidate the role of *GbSER02* in regulating cotton fiber development, a comparative transcriptome analysis was performed using 10 DPA fibers from the *GbSER02*‐OE lines and control. Using a significance threshold of *P_adj_* ≤ 0.01, we identified 164 differentially expressed genes, including 137 upregulated and 27 downregulated genes. Gene Ontology (GO) analysis showed that genes related to cellular carbohydrate metabolism were significantly upregulated in the OE lines (**Figure** **6**a). The xyloglucan endotransglucosylase/hydrolase (*XTH2*, *GhM_D02G0203*) gene involving in cell wall remodeling^[^ ^32^ ^]^ was found among the enriched genes (Table S2, Supporting Information). qRT‐PCR assay confirmed that *GhXTH2* expression was upregulated, consistent with the transcriptome results (Figure 6b). Previous studies have shown that *XTH* regulates fiber cell elongation^[^ ^33^, ^34^, ^35^ ^]^ and is induced by GA.^[^ ^36^ ^]^ In addition to *XTH*, α‐expansin (EXPA) is another key cell wall protein that facilitates cell wall loosening,^[^ ^33^, ^37^ ^]^ and also induced by GA.^[^ ^6^ ^]^ Then, a quantitative analysis of the *GhEXPA* genes was conducted in cotton, determining that *GhEXPA1* (*GhM_D12G2171*) was significantly upregulated in the fibers of *GbSER02*‐OE cotton (Figure 6c). Moreover, based on the transcriptomic data, at the elongation stage, *XTH2* and *EXPA1* exhibited higher expression levels in *G. barbadense* than in *G. hirsutum* (Figure 6d,e), which is consistent with the functions of these genes in fiber elongation. These results indicated that *GbSER02* controlled fiber elongation by positively regulating *XTH2* and *GhEXPA1* expression.

Based on untargeted metabolomic data from the 10 DPA fibers of the control and *GbSER02*‐OE cotton lines and the results of a Kyoto Encyclopedia of Genes and Genomes (KEGG) pathway analysis, flavonoid synthesis was primarily enriched in the *GbSER02*‐OE lines (Figure 6f). Among the flavonoids, prunin (a derivative of naringenin) was significantly reduced in the *GbSER02*‐OE lines (Figure 6g). qRT‐PCR analysis of flavonoid metabolism‐related genes revealed that *GhCHS1 (GhM_A02G0287)*, the first key gene in the pathway, was markedly downregulated in the *GbSER02*‐OE lines, which correlated with a substantially reduced flavonoid content compared to that in the control (Figure 6h,i). Transcriptome data from fiber tissues between *G. barbadense* and *G. hirsutum* revealed significantly lower *CHS1* expression levels in *G. barbadense* than in *G. hirsutum* at the fiber elongation stage (Figure 6j). Considering the above results, as well as GA_3_ inhibits flavonoid biosynthesis,^[^ ^38^ ^]^ and flavonoid‐related compounds negatively affect fiber length,^[^ ^39^ ^]^ we inferred that GbSER02 positively regulated GA contents by interacting with GhVOZ1. This promoted the expression of genes related to cell wall loosening and flavonoid metabolism, ultimately improving fiber length.

## 3 Discussion

The elongation period of cotton fiber development is crucial for ensuring fiber yield and quality. Therefore, it is necessary to identify the genes that influence fiber elongation. In plants, serpin is multifunctional that exhibits inhibitory activity, and is instrumental in regulating cell death in response to bacterial, fungal, and abiotic stresses and defending against insect predation.^[^ ^20^, ^40^, ^41^, ^42^ ^]^ However, the functions and regulatory mechanisms of serpins in cotton fiber development require elucidation. In the present study, we found that *SER02* was significantly differentially expressed during fiber elongation between *G. barbadense* and *G. hirsutum*. SNP^517th^ on chromosome A11 of *G. hirsutum* caused premature termination of the serpin sequence, resulting in the formation of the truncated GhSER02, which lacks the RCL structural domain. Disrupted genes accumulate more rapidly in allopolyploid cotton than in diploid cotton, and these mutated genes tend to have lower expression levels than conserved genes, which affects their function.^[^ ^43^, ^44^ ^]^ In this study, *GbSER02* or *GhSER02′* overexpression promoted cell elongation in *Arabidopsis*, whereas *GhSER02* overexpression did not, indicating that the SNP^517th^ mutation led to functional differences between *GbSER02* and *GhSER02*. Furthermore, *GbSER02* overexpression in *G. hirsutum* significantly promoted fiber elongation. These findings demonstrate that SNP^517th^ is a crucial site for *SER02* in regulating fiber length in cotton.

GAs play a crucial role in the development of cotton fiber cells, either by regulating the expression of downstream genes or by interacting with other plant hormones.^[^ ^6^, ^7^, ^45^ ^]^ However, the relationship between GbSER02 and GAs during fiber development is unclear. In the present study, we employed multiple approaches to validate the interaction between GbSER02 and transcriptional factor GhVOZ1. This interaction is distinct from that of AtSerpin1, which interacts with protease RD21. Furthermore, we demonstrated that both GbSER02 and GhSER02 interacted with GhVOZ1. However, in the 10 DPA fibers, the expression level of *GbSER02* was significantly higher than that of *GhSER02*. Previous studies have indicated that truncated proteins resulting from premature termination codons (PTCs) triggers nonsense‐mediated mRNA decay, further reducing protein expression.^[^ ^46^, ^47^ ^]^ These findings indicate that the PTC of *GhSER02* might lead to protein reduction, thereby compromising its function. In higher plants, *VOZ1* regulates various biological processes, including flower induction, plant growth and development, and responses to environmental stress.^[^ ^30^, ^48^, ^49^ ^]^ We confirmed that GbSER02 interacted with GhVOZ1 in the cytoplasm using a BiFC assay. Subcellular localization assay indicated that GhVOZ1 was localized in the cytoplasm, which was consistent with the localization of AtVOZ2 in *Arabidopsis*.^[^ ^50^ ^]^ Thus, we speculated that GbSER02 retained GhVOZ1 in the cytoplasm to inhibit its activity. However, to function as a transcription factor, GhVOZ1 must enter the nucleus. Future studies should investigate when and how GhVOZ1 enters the nucleus. We also found that *GhVOZ1* directly bound to the promoter of *GhGA3ox1* and repressed its transcription, and *GbSER02* overexpression in cotton increased the GA_3_ content of the fibers. Thus, the GbSER02‐GhVOZ1‐GhGA3ox1 module promoted GA_3_ biosynthesis and facilitated fiber elongation.

Genes related to cell wall loosening are essential to the rapid elongation of fiber cells. Application of exogenous GA to cultured ovules enhances fiber length and stimulates *XTH* expression,^[^ ^36^ ^]^ thereby facilitating the relaxation of cell walls, promoting fiber elongation.^[^ ^35^ ^]^ Consistent with previous studies, *GbSER02* overexpression increased the GA₃ content in cotton fibers, upregulating *GhXTH2* expression, suggesting that *GhXTH2* operated downstream of GA signaling. We verified that *GhEXPA1* was significantly expressed in the fibers of GbSER02‐OE cotton and that the accumulation of flavonoid‐related compounds, especially naringenin, negatively affected fiber elongation.^[^ ^51^ ^]^ Consistent with these findings, *GhCHS1* was significantly downregulated and flavonoid content was significantly decreased in *GbSER02*‐OE cotton with longer fibers, indicating that fewer flavonoid compounds benefited fiber elongation. Due to the negative regulation of flavonoid biosynthesis by GA in leaves,^[^ ^38^ ^]^ increased GA levels were verified to induce a decrease in flavonoid metabolites in the fibers of *GbSER02*‐OE cotton.

Transcriptome data from GbSER02‐OE lines also revealed the significant enrichment of upregulated genes related to antioxidant, oxidoreductase, and peroxidase activity, which most belong to the peroxidase gene family. Peroxidases are also involved in lignin biosynthesis.^[^ ^52^, ^53^, ^54^ ^]^ *WLIM1a* mediates an increase in lignin and lignin‐derived phenolic compounds, which improves fiber fineness.^[^ ^55^ ^]^ Thus, the overexpression of *GbSER02* may enhance lignin deposition in fibers, leading to an improvement in fiber fineness. Although higher lignin content was associated with fiber shortening in some studies,^[^ ^54^, ^56^ ^]^ our results suggested that increased expression of cell wall loosening‐related genes in the overexpression lines contributed to cell wall remodeling. This process may alleviate the inhibitory effect of lignin on fiber elongation, ultimately resulting in longer and finer fibers.

## 4 Conclusion

**Figure 7:**
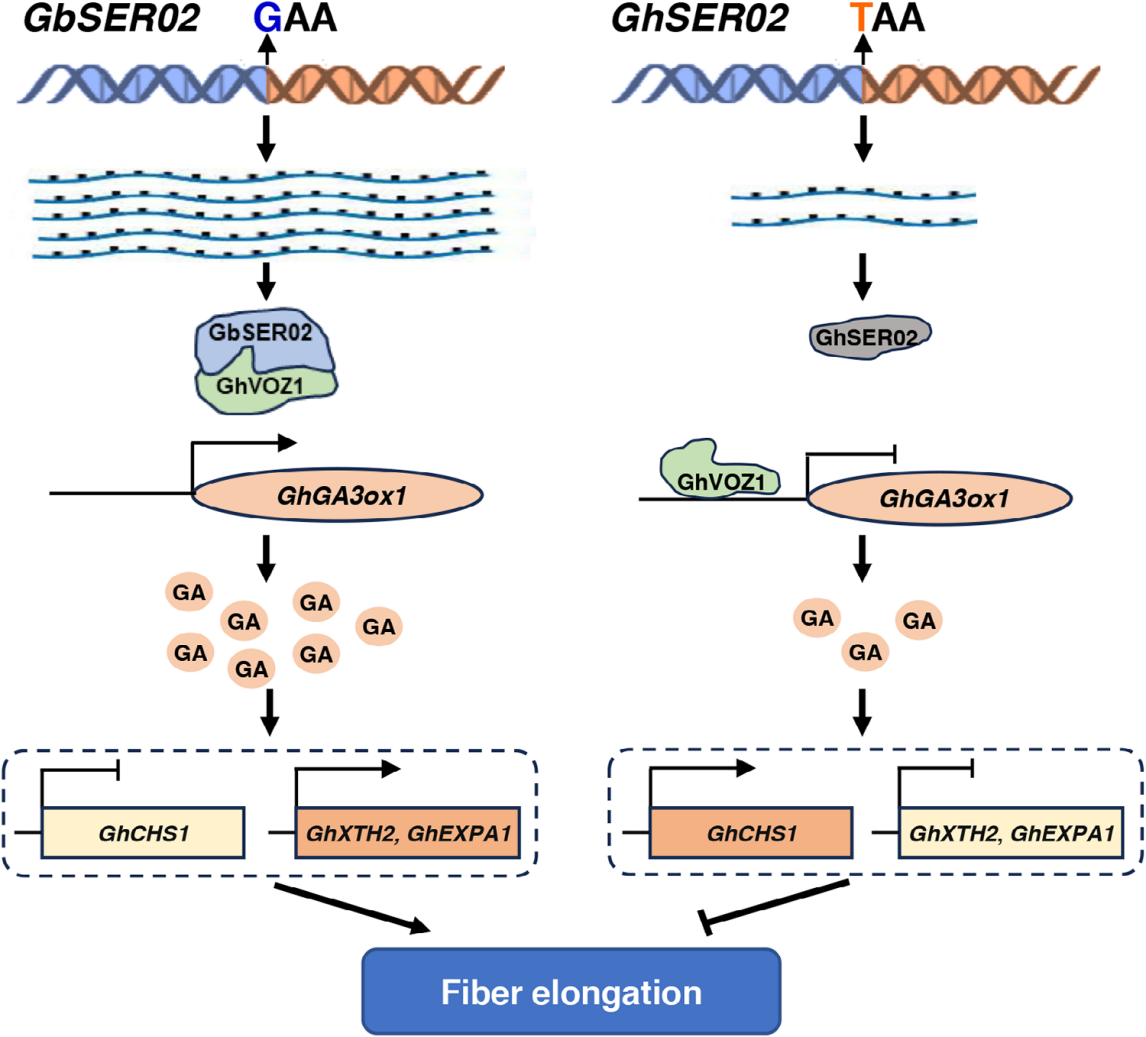
Model showing that the GbSER02‐GhVOZ1‐GhGA3ox1 module positively regulates fiber elongation.

This study proposed a model to illustrate the role of *GbSER02* in regulating cotton fiber elongation (**Figure** **7**). In this model, the high expression of *GbSER02* in *G. barbadense* or its overexpression in *G. hirsutum* produces abundant GbSER02 protein. This protein then interacts with and binds most of the GhVOZ1 in the cytoplasm, thereby alleviating the inhibitory effect of GhVOZ1 on *GhGA3ox1* and promoting GA_3_ biosynthesis. Subsequently, GA_3_ induces the expression of cell wall loosening‐related genes and decreases the expression of flavonoid synthesis‐related genes, ultimately facilitating fiber elongation. However, for *G. hirsutum*, low expression of *GhSER02* and the truncation of its protein increase GhVOZ1 binding to the promoter of *GhGA3ox1*, which inhibits its transcription and results in shorter fibers. Thus, this study provides novel insights into the mechanisms of fiber development and offers reliable molecular markers for improving fiber quality in cotton.

## 5 Experimental Section

### Identification of the Serpin Family in Cotton Species and Sequence Alignment

The genome sequences and annotation data for *G. hirsutum* (HEBAU), *G. barbadense* (HEBAU), *G. arboreum* (CRI), and *G. raimondii* (JGI) were downloaded from CottonFGD (http://www.cottonfgd.org/),^[^ ^57^, ^58^, ^59^ ^]^ while those for *G. tomentasum* (HGS), *G. mustelinum* (JGI) and *G. darwinii* (HGS) were downloaded from CottonMD (https://yanglab.hzau.edu.cn/CottonMD/org/). The Hidden Markov Model (HMM) database, specifically, the Pfam database (http://pfam.xfam.org/), was used to download the conserved structural domain of serpins (SerpinP‐plants, cd02043). The *serpin* genes of cotton species were identified using the Simple HMM search function of TBtools software (V2.119).^[^ ^60^ ^]^ The sequences of serpin family members were assessed to exclude incomplete sequences of conserved structural domains on the National Center for Biotechnology Information Conserved Domains website (https://www.ncbi.nlm.nih.gov/Structure/cdd/wrpsb.cgi). The protein sequence of *A. thaliana* VOZ2 was retrieved from the Arabidopsis Information Resource (http://www.arabidopsis.org/), while the sequence of tomato VOZ1 was obtained from Phytozome version 12.1 (https://phytozome.jgi.doe.gov/pz/portal.html). The protein sequences are listed in Table S3 (Supporting Information). Sequence alignment was performed using ClustalX 1.83,^[^ ^61^ ^]^ and the chromosomal locations of the serpin family members were visualized using the Gene Location Visualize function from GTF/GFF in TBtools (V2.119).

### Recognition of the Ribosome Scanning Sequence

Eukaryotic mRNA has a “CCACC” ribosome‐scanning sequence in the upstream of the AUG start codon, which serves as the signal for ribosome recognition of the first AUG. By using sequence alignment methods, if a region similar to the known ribosome scanning sequence is found near the start codon of the gene, then that region may be identified as a ribosome‐scanning sequence.

### Plant Materials and Growth Condition

*G. barbadense* line Pima90 and *G hirsutum* cultivar NDM8 were used for gene cloning and qRT‐PCR assay. The *G. hirsutum* cultivar “Jin668,” which was provided by Huazhong Agricultural University Prof. Xianlong Zhang, was selected as the transgenic receptor. Jin668 and transgenic *GbSER02* plants were grown in a greenhouse at 60% humidity, with a 16 h light/8 h dark cycle and temperature of 30 °C/25 °C. Cotton bolls were harvested at 10 DPA from both non‐transgenic control and transgenic plants, and then the fibers were collected and stored at −80 °C prior to use.

### Arabidopsis Transformation and Phenotypic Observation

The constructs of *GhSER02*, *GhSER02′*, and *GbSER02* was introduced into a pGreen vector with 6HA tag and transformed them into *Arabidopsis* (Columbia‐0) using the floral dip method. These plants were grown in a growth chamber under long‐day conditions (16 h light/8 h dark, 22 °C, ≈70% relative humidity). Transgenic lines were selected based on herbicide resistance and PCR identification, and homozygous lines were generated by self‐pollination over two generations.

The fifth or sixth rosettes from the transgenic *Arabidopsis* and WT plants were collected before bolting. For each line, nine–ten leaves were sampled and decolorized in absolute ethanol until all green pigment was removed. A 50% glycerol solution was prepared for leaf storage. The leaves were then placed on a microscope slide with the adaxial surface facing upward. Epidermal hairs located away from the leaf veins and edges were counted, observed, and photographed using an optical microscope (Olympus BX51, Japan). Excess of 350 epidermal hairs were counted for the WT control and transgenic lines of *GhSER02*‐OE, *GhSER02′*‐OE, and *GbSER02*‐OE. Finally, epidermal hair lengths were measured using ImageJ software.

After *Arabidopsis* seeds were disinfected, they were evenly placed on 1/2 MS medium for cultivation under light conditions. After 10 d, root lengths were measured. *Arabidopsis* roots were observed and photographed using an optical microscope for statistical analysis. The roots were then cut using a scalpel, and the root cell lengths were observed and photographed under a confocal microscope for further analysis. Finally, the root and root cell lengths were measured using ImageJ software.

### Cotton Transformation and Phenotypic Observation

The CDS of *GbSER02* was cloned into the pCambia2301 vector driven by the Ubi promoter to construct an overexpression vector. The primer sequences are listed in Table S4 (Supporting Information). The overexpression vector was transformed into *Agrobacterium tumefaciens* strain GV3101, and *Agrobacterium*‐mediated cotton transformation was performed as previously described.^[^ ^62^ ^]^ After selection based on kanamycin resistance and PCR amplification, the T_2_ generation of the transgenic cotton was used for phenotypic analysis. Cotton plants were cultivated under natural conditions until the bolls opened, and then the fibers were harvested. The harvested fibers were used for fiber length measurements. Overall, 10–15 g of fibers for each line was measured using an HVI1000 instrument. The harvested mature fibers (Control, OE‐1, and OE‐2) were embedded in resin and sectioned using a slicer (Leica HistoCore NANOCUT, Germany) and observed under an optical microscope (Olympus BX51, Japan). The cell wall thickness was subsequently measured, and >30 fiber cells from each transgenic line were investigated.

### qRT‐PCR Analysis

RNA was extracted from 10 DPA fiber tissues from both the control and transgenic *GbSER02* cotton lines using an RNA extraction kit (TianGen, China). Two micrograms of total RNA was used for first‐strand cDNA synthesis with the Superscript First‐Strand Synthesis System (TransGen Biotech Co., China). Transcript abundance was quantified by qRT‐PCR with the SYBR Green Master Mix (2×; TransGen Biotech Co., China). The qRT‐PCR assay program was as follows: 95 °C for 10 min, followed by 40 cycles of 95 °C for 10 s and 60 °C for 30 s. Cotton *Histone3* (GenBank accession no. AF024716) was used as an internal control. qRT‐PCR was independently conducted in triplicate. For *SER02*, we designed quantitative primers located upstream of the SNP^517th^ site to analyze the differences in expression between *G. barbadense* and *G. hirsutum*.

### Subcellular Localization

The full‐length ORFs of *GbSER02* and *GhVOZ1* were inserted into a GFP tag vector. Subsequently, *GbSER02‐GFP* and *GhVOZ1‐GFP* constructs were transformed into *A. tumefaciens* strain GV3101, whose cells were collected and suspended in the resuspending solution (10 mM MgCl_2_, 10 mM 2‐Morpholinoethanesulfonic acid, and 200 µM acetosyringone). After 3 h of culture at 25 °C, the suspension was infiltrated into the leaves of *N. benthamiana*. GFP fluorescence was observed 2 d later using a confocal microscope (Zeiss LSM‐710, Germany).

### Luciferase Complementation Imaging (LCI) Assay

The CDS of *GbSER02* and *GhVOZ1* were fused with pCAMBIA1300‐nLUC and pCAMBIA1300‐cLUC, respectively. The fusion plasmids were co‐transformed into *A. tumefaciens* strain GV3101 for transient expression in *N. benthamiana* leaves. Following infiltration, the plants were grown in the darkness for 1 d, followed by 2 d of light conditions. LUC activity was measured using a chemiluminescence detector (Tanon 5200 Multi, China) imaging system.

### For Co‐IP Assay

The full‐length ORFs of *GbSER02* and *GhVOZ1* were cloned into the PS1300TB‐bHA and PS1300TB‐bFlag vectors, respectively. The constructs were transformed into *Agrobacterium* strain GV3101, and appropriate pairs of *Agrobacterium* cells were infiltrated into the leaves of *N. benthamiana*. After 2–3 d of infiltration, total proteins from *N. benthamiana* leaves were extracted with lysate (100 mM Tris‐HCl pH 8.0, 100 mM NaCl, 500 mM sucrose, 10 mM EDTA, 0.1% sodium deoxycholate, 0.3% Triton X‐100, 0.1% CHAPS, 0.3% CAPSO, and protease inhibitor mix). A small amount of lysate was collected and used for the other experiments, and the HA antibody (10 µg) was added to the remaining lysate, which was incubated overnight at 4 °C. Pierce Protein A/G Agarose Beads (Thermo Fisher Scientific, USA) were washed repeatedly with the lysis buffer. The pretreated beads were added to the cell lysate, incubated overnight at 4 °C, and centrifuged at 600 ×g for 3 min at 4 °C. Then, the supernatant was removed, and the agarose beads were washed three times with 1 mL lysis buffer. Then, 100 µL of 2× sodium dodecyl sulfate loading buffer was added. The samples were then incubated at 95 °C for 5 min and analyzed using immunoblotting with anti‐HA and anti‐Flag antibodies.

### BiFC Assay

The full‐length coding sequences of *GbSER02* and *GhVOZ1* were fused with pSAT1‐nEYFP‐N1 and pSAT1‐nEYFP‐C1, respectively. Recombinant plasmids were introduced into cotton protoplasts. YFP fluorescence was observed 2 d after co‐infiltration using a confocal microscope (Zeiss LSM710, Germany). Cotton protoplasts were prepared as follows: After germinating the cotton seeds, the seedlings were hydroponically cultivated for 3 d. Young roots were then harvested, sectioned into thin slices, and subjected to enzymatic digestion to isolate the protoplasts.

### Y2H Assay

The CDS of *GbSER02* and *GhVOZ1* were inserted into the pGBKT7 vector (bait vector) and pGADT7 vector (prey vector), respectively. The recombinant plasmids were transformed into the yeast strain Y2HGold using the lithium acetate transformation procedure. In total, 10 µL of transformed yeast cells were spotted onto synthetic defined (SD) medium without leucine, tryptophan, and histidine, and then incubated at 30 °C for 3–5 d.

### Y1H Assay

For the Y1H assay, a 235 bp (from −1985 bp to −2219 bp) promoter fragment of *GhGA3ox1* was cloned and inserted into the pAbAi vector (Coolaber, China). The coding sequence of *GhVOZ1* was then inserted into the pGADT7 vector (Clontech, USA), and the recombinants were transformed into yeast strain Y1HGold. A total of 10 µL transformed yeast cells were spotted onto SD medium with leucine, supplemented with AbA (1,000 ng mL^−1^), and then incubated at 30 °C for 3–5 d.

### EMSA Assay

A 5′‐biotinylated oligonucleotide (5′‐CAGTAGAACGCTCTCAAC‐3′) was used as the probe, which was incubated with nuclear extract at 25 °C for 30 min. The entire reaction mixture was run on a non‐denaturing 6% polyacrylamide gel in 0.5× Tris‐Borate‐EDTA buffer at 60 V and 4 °C for 1 h and then transferred onto Biodyne B nylon membranes (Pall Corporation, USA). Signals were visualized using the reagents included in the kit and ChemiDoc XRS (Bio‐Rad Laboratories, USA).

### Dual‐LUC Assay

For the dual‐LUC assays, the promoter fragment (P235) of *GhGA3ox1* was amplified from *G. hirsutum* NDM8 and inserted upstream of the firefly LUC reporter gene of the pGreenII 0800‐LUC vector. The *GhVOZ1* gene was constructed into the pGreenII 62sk vector as an effector. The recombinant vectors were transformed into *A. tumefaciens* strain GV3101 and transiently co‐infiltrated into the leaf epidermal cells of *N. benthamiana*. LUC activity was measured using a chemiluminescence detector (Tanon 5200 Multi, China) imaging system.

### GA and Flavonoid Content Determination and Untargeted Metabolomic Assay

Cotton fibers (10 DPA) from the *GbSER02*‐OE and control lines were ground thoroughly into a powder in liquid nitrogen. The samples were then sent to ProNet Biotech Co., Ltd. (China) for GA (GA_1_, GA_3_, and GA_4_) determination and Oebiotech Co., Ltd. (China) for untargeted metabolomic assay. The flavonoid content was detected with the Plant Flavonoids Content Assay Kit (Boxbio AKPL015C, China).

### Virus‐Induced Gene Silencing (VIGS)

A specific nucleotide acid sequence from *GhVOZ1* was inserted into the tobacco rattle virus binary vector pYL156. *GhCLA1* was used as a control to assess the silencing efficiency as it could induce a photobleaching phenotype. The VIGS assay was performed as previously described.^[^ ^63^ ^]^

### Statistical Analysis

Two‐tailed Student's *t*‐test was used to compare the differences in gene expression and fiber length. Tukey's honestly significant difference (HSD) test was used to compare the trichome length, root length, and root cell length between the WT and overexpression lines in *Arabidopsis*. The statistical methods used in this study are described in the corresponding figure legends. All statistical analyses were performed using GraphPad Prism 8 software (https://www.graphpad.com/scientific‐software/prism/).

## Conflict of Interest

The authors declare no conflicts of interest.

## Author Contributions

H.J. D.Z., and Q.G. contributed equally to this work. H.J., D.Z., and Q.G. performed the experiments. Z.S. and H.K. contributed to fiber quality in the CSSL population. Y.L., J.L., and W.L. contributed to fiber sample preparation. H.J. wrote the manuscript with the support of Y.Z., X.W., and Z.M. X.W., Z.M. and Y.Z. conceived the original study and supervised the project. All authors have read and approved the manuscript.

## Supporting information



Supporting Information

Supporting Information

## Data Availability

The data that support the findings of this study are available in the supplementary material of this article.

## References

[1] H. J. Kim , B. Triplett , Plant Physiol. 2001, 127, 1361. 11743074 PMC1540163

[2] G. Huang , J. Q. Huang , X. Y. Chen , Y. X. Zhu , Annu. Rev. Plant Biol. 2021, 72, 437. 33428477 10.1146/annurev-arplant-080720-113241

[3] C. H. Haigler , L. Betancur , M. R. Stiff , J. R. Tuttle , Front. Plant Sci. 2012, 3, 104. 22661979 10.3389/fpls.2012.00104 PMC3356883

[4] M. Jan , Z. Liu , C. Guo , X. Sun , Int. J. Mol. Sci. 2022, 23, 5004. 35563394 10.3390/ijms23095004 PMC9101851

[5] Y. H. Xiao , D. M. Li , M. H. Yin , X. B. Li , M. Zhang , Y. J. Wang , Y. Pei , Plant Physiol. 2010, 167, 829. 10.1016/j.jplph.2010.01.003 20149476

[6] C. M. Shan , X. X. Shangguan , B. Zhao , X. F. Zhang , L. M. Chao , C. Q. Yang , X. Y. Chen , Nat. Commun. 2014, 5, 5519. 25413731 10.1038/ncomms6519 PMC4263147

[7] Z. L. Tian , Y. Z. Zhang , L. P. Zhu , B. Jiang , H. Q. Wang , R. X. Gao , G. H. Xiao , Plant Cell 2022, 34, 4816. 36040191 10.1093/plcell/koac270 PMC9709996

[8] P. He , L. Zhu , X. Zhou , X. Fu , Y. Zhang , P. Zhao , B. Jiang , H. Wang , G. H. Xiao , Dev. Cell. 2024, 59, 723. 38359829 10.1016/j.devcel.2024.01.018

[9] Z. W. Liu , Z. W. Sun , H. F. Ke , B. Chen , Q. S. Gu , M. Zhang , N. Wu , L. T. Chen , Y. B. Li , C. S. Meng , G. N. Wang , L. Q. Wu , G. Y. Zhang , Z. Y. Ma , Y. Zhang , X. F. Wang , Int. J. Mol. Sci. 2023, 24, 8293.37175999

[10] B. Goopu , D. A. Lomas , Annu. Rev. Biochem. 2009, 78, 147. 19245336 10.1146/annurev.biochem.78.082107.133320

[11] G. A. Silverman , D. A. Lomas , Annu. Rev. Biochem. 2007, 280, 13735.

[12] T. H. Roberts , J. Hejgaard , Funct. Integr. Genomics 2008, 8, 1. 18060440 10.1007/s10142-007-0059-2

[13] J. A. Huntington , R. J. Read , R. W. Carrell , Nature 2000, 407, 923. 11057674 10.1038/35038119

[14] F. C. Peterson , N. C. Gordon , P. G. Gettins , Biochemistry 2000, 39, 11884. 11009600 10.1021/bi001152+

[15] J. Sun , J. C. Whisstock , P. Harriott , B. Walker , A. Novak , P. E. Thompson , P. I. Bird , Biochemistry 2001, 276, 15177. 10.1074/jbc.M006645200 11278311

[16] M. Wilczynska , M. Fa , P. I. Ohlsson , T. Ny , Biochemistry 1995, 270, 29652. 10.1074/jbc.270.50.29652 8530349

[17] S. W. Dahl , S. Rasmussen , J. Hejgaard , Biochemistry 1997, 271, 25083. 10.1074/jbc.271.41.25083 8810262

[18] N. Lampl , O. Budai‐Hadrian , O. Davydov , T. V. Joss , S. J. Harrop , P. M. G. Curmi , R. Fluhr , Biochemistry 2010, 285, 13550. 10.1074/jbc.M109.095075 PMC2859516 20181955

[19] L. Bhattacharjee , P. K. Singh , S. Singh , A. K. Nandi , Plant Biol 2015, 58, 327.

[20] R. Dhanushkodi , C. Matthew , M. T. McManus , P. Dijkwel , New Phytol 2018, 220, 196. 29974467 10.1111/nph.15298

[21] M. Cohen , T. H. Roberts , R. Fluhr , Annu. Rev. Plant Biol. 2015, 15, 28.

[22] H. Ostergaard , S. K. Rasmussen , T. H. Roberts , J. Hejgaard , Biochemistry 2000, 275, 33272. 10.1074/jbc.M004633200 10874043

[23] J. Hejgaard , FEBS. Lett. 2001, 488, 149. 11163762 10.1016/s0014-5793(00)02425-x

[24] S. H. Ke , G. S. Coobms , K. Tachias , M. Navre , D. R. Corey , E. L. Madison , Biochemistry 1997, 272, 16603. 10.1074/jbc.272.26.16603 9195973

[25] M. I. Plotnick , N. M. Schechter , Z. M. Wang , X. Liu , H. Rubin , Biochemistry 1997, 36, 14601. 9398179 10.1021/bi971530j

[26] C. A. Ibarra , G. E. Blouse , T. D. Christian , J. D. Shore , Biochemistry 2004, 279, 3643. 10.1074/jbc.M310601200 14594804

[27] X. Y. Guan , N. Yu , X. X. Shang Guan , S. Wang , S. Lu , L. J. Wang , Chin. Sci. Bull. 2007, 52, 1734.

[28] X. Guan , M. Pang , G. Nah , X. Shi , W. Ye , D. M. Stelly , Z. J. Chen , Nat. Commun. 2014, 5, 5519. 24430011 10.1038/ncomms4050

[29] Y. Yu , S. Wu , J. Nowak , G. Wang , Z. Kong , Nat. Plants 2019, 5, 498. 31040442 10.1038/s41477-019-0418-8

[30] D. Luo , L. Qu , M. Zhong , X. Li , H. Wang , J. Miao , X. Zhao , Biosci. Biotechnol. Biochem. 2020, 84, 1384. 32186471 10.1080/09168451.2020.1740971

[31] L. Chong , R. Xu , P. C. Huang , P. C. Guo , M. K. Zhu , H. Du , X. L. Sun , L. X. Ku , J. K. Zhu , Y. F. Zhu , Plant Cell 2001, 34, 2022. 10.1093/plcell/koac026 PMC9048945 35099557

[32] J. M. Eklöf , H. Brumer , Plant Physiol. 2010, 153, 456. 20421457 10.1104/pp.110.156844 PMC2879796

[33] D. J. Cosgrove , Nat. Rev. Mol. Cell. Biol. 2005, 6, 850. 16261190 10.1038/nrm1746

[34] J. Lee , T. H. Burns , G. Light , Y. Sun , M. Fokar , Y. Kasukabe , R. D. Allen , Planta 2010, 232, 1191. 20711605 10.1007/s00425-010-1246-2

[35] M. Y. Shao , X. D. Wang , M. Ni , N. Bibi , S. N. Yuan , W. Malik , W. H. P. Zhang , Y. X. Liu , S. J. Hua , Genet. Mol. Res. 2011, 10, 3771. 22057988 10.4238/2011.October.27.1

[36] L. Aleman , J. Kitamura , H. Abdel‐Mageed , J. Lee , Y. Sun , M. Nakajima , Plant Mol. Biol. 2008, 68, 1. 18506581 10.1007/s11103-008-9347-z

[37] J. S. McQueen‐Maon , D. J. Cosgrove , Plant Physiol. 1995, 107, 87. 11536663 10.1104/pp.107.1.87 PMC161171

[38] H. Sun , H. Cui , J. Zhang , J. Kang , Z. Wang , M. Li , F. Li , Q. Yang , R. Long , Int. J. Mol. Sci. 2021,22, 9291. 34502200 10.3390/ijms22179291 PMC8431309

[39] S. Hua , X. Wang , S. Yuan , M. Shao , X. Zhao , S. Zhu , L. Jiang , Crop Sci. 2007, 47, 1540.

[40] N. Lampl , N. Alkan , O. Davydov , R. Fluhr , Plant J. 2013, 74, 498. 23398119 10.1111/tpj.12141

[41] L. Bhattacharjee , P. K. Singh , S. Singh , A. K. Nandi , Plant Biol 2015, 58, 327.

[42] E. T. Johnson , C. D. Skory , T. A. Naumann , M. A. Jairajpuri , P. F. Dowd , Agri. Gene 2016, 2, 11.

[43] J. L. Conover , J. F. Wendel , Mol. Biol. Evol. 2022, 39, 24. 10.1093/molbev/msac024 PMC8841602 35099532

[44] L. Fang , Z. Y. Zhang , T. Zhao , N. Zhou , H. Mei , X. Q. Huang , F. Wang , Z. F. Si , Z. G. Han , S. Lu , Y. Hu , X. Y. Guan , T. Z. Zhang , Plant Biotechnol. J. 2022, 20, 1770. 35633313 10.1111/pbi.13862 PMC9398350

[45] W. Q. Bai , Y. H. Xiao , J. Zhao , S. Q. Song , L. Hu , J. Y. Zeng , Y. Pei , PLoS One 2014, 9, 96537. 10.1371/journal.pone.0096537 PMC4015984 24816840

[46] F. He , A. Jacobson , Annu. Rev. Genet. 2015,49, 339. 26436458 10.1146/annurev-genet-112414-054639 PMC4837945

[47] S. Lykke‐Andersen , T. H. Jensen , Nat. Rev. Mol. Cell. Biol. 2015, 16, 665. 26397022 10.1038/nrm4063

[48] N. Mitsuda , T. Hisabori , K. Takeyasu , M. H. Sato , Plant Cell Physiol. 2004, 45, 845. 15295067 10.1093/pcp/pch101

[49] Y. Nakai , S. Fujiwara , Y. Kubo , M. H. Sato , Plant Signal Behav 2013, 8, 23358. 10.4161/psb.23358 PMC3676503 23299334

[50] S. Devarshi , M. Anna , J. W. Gillikin , M. H. Sato , T. A. Long , Plant Cell Environ 2018, 41, 2463. 29878379 10.1111/pce.13363

[51] J. Tan , L. Tu , F. Deng , H. Hu , Y. Nie , X. Zhang , Plant Physiol. 2023, 162, 86. 10.1104/pp.112.212142 PMC3641232 23535943

[52] K. A. Blee , J. W. Choi , A. P. O'Connell , W. Schuch , N. G. Lewis , G. P. Bolwell , Phytochemistry 2003, 64,163. 12946415 10.1016/s0031-9422(03)00212-7

[53] M. N. Quiroga , C. Guerrero , M. A. Botella , A. Barceló , I. Amaya , M. I. Medina , V. Valpuesta , J. Biol. Chem. 2017, 122, 1119. 10.1104/pp.122.4.1119 PMC58946 10759507

[54] Z. Y. Gao , W. J. Sun , J. Wang , C. Y. Zhao , K. J. Zuo , Plant Sci 2019, 286, 7. 31300144 10.1016/j.plantsci.2019.05.020

[55] L. B. Han , Y. B. Li , H. Y. Wang , X. M. Wu , C. L. Li , M. Luo , S. J. Wu , Z. S. Kong , Y. Pei , G. L. Jiao , G. X. Xia , Plant Cell 2013, 25, 4421. 24220634 10.1105/tpc.113.116970 PMC3875727

[56] H. P. Li , J. G. Guo , K. Li , Y. W. Gao , H. Li , L. Long , Z. C. Chu , Y. B. Du , X. L. Zhao , B. Zhao , C. Lan , J. R. Botella , X. B. Zhang , K. P. Jia , Y. C. Miao , Plant J. 2024,120, 2846. 39559968 10.1111/tpj.17149

[57] K. B. Wang , Z. W. Wang , F. G. Li , W. W. Ye , J. Y Wang , G. L. Song , S. X. Yu , Nat. Genet. 2012, 44, 1098.22922876

[58] F. G. Li , G. Y. Fan , K. B. Wang , F. M. Sun , Y. L. Yuan , G. L. Song , S. X. Yu , Nat. Genet. 2014, 46, 567.24836287

[59] Z. Y. Ma , Y. Zhang , L. Q. Wu , G. Y. Zhang , Z. W. Sun , Z. K. Li , Y. F. Jiang , Nat. Genet. 2021, 53, 1385. 34373642 10.1038/s41588-021-00910-2 PMC8423627

[60] C. J. Chen , H. Chen , Y. Zhang , H. R. Thomas , M. H. Frank , Y. H. He , R. Xia , Mol. Plant. 2020, 13, 1194. 32585190 10.1016/j.molp.2020.06.009

[61] R. Chenna , H. Sugawara , T. Koike , R. Lopez , T. J. Gibson , D. G. Higgins , J. D. Thompson , Nucleic Acids Res. 2003, 31, 3497. 12824352 10.1093/nar/gkg500 PMC168907

[62] F. F. Li , S. J. Wu , T. Z. Chen , J. Zhang , H. H. Wang , W. Z. Guo , O. Culture , Plant Sci. 2009, 97, 225.

[63] X. Q. Gao , T. Wheeler , Z. H. Li , C. M. Kenerley , P. He , L. Shan , Plant J. 2011, 66, 293. 21219508 10.1111/j.1365-313X.2011.04491.x PMC3078967

