# Supplementary material for: GbSER02 Interacts With GhVOZ1 to Promote Fiber Elongation by Modulating Gibberellin Synthesis in Cotton
Source: Adv Sci (Weinh). 2025 Jun 25;12(35):e17578. doi: 10.1002/advs.202417578 (PMC12463063; doi:10.1002/advs.202417578)
Supplement: Supplementary file 1 — Supporting Information [file ADVS-12-e17578-s001.docx]

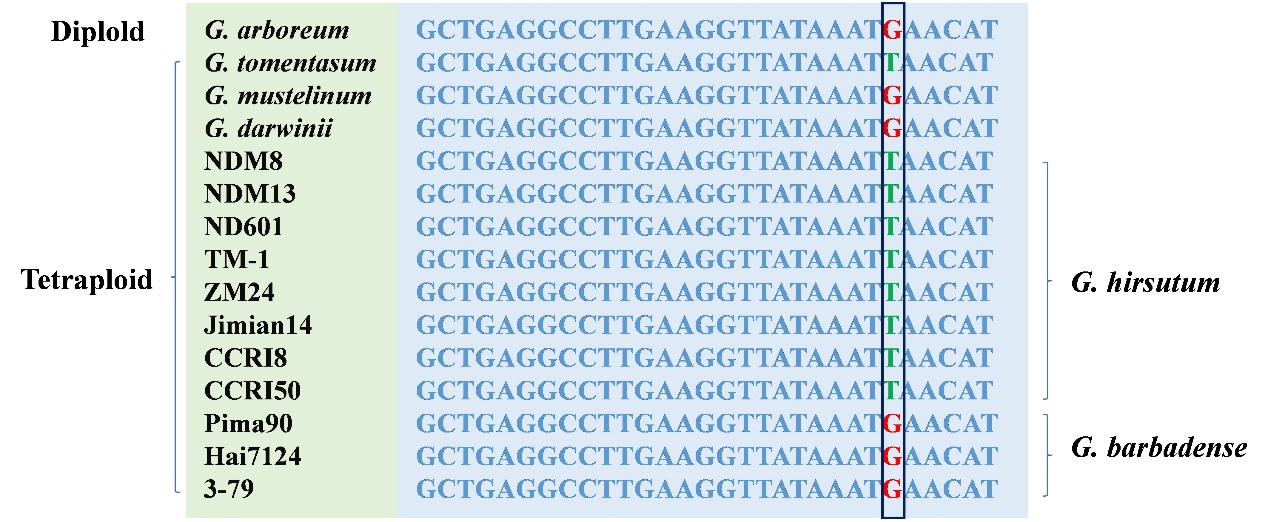


**Supplementary Fig.1 Genotype of serpins on chromosome A11 across different *Gossypium* spp.** The black box contains the key SNP G/T.


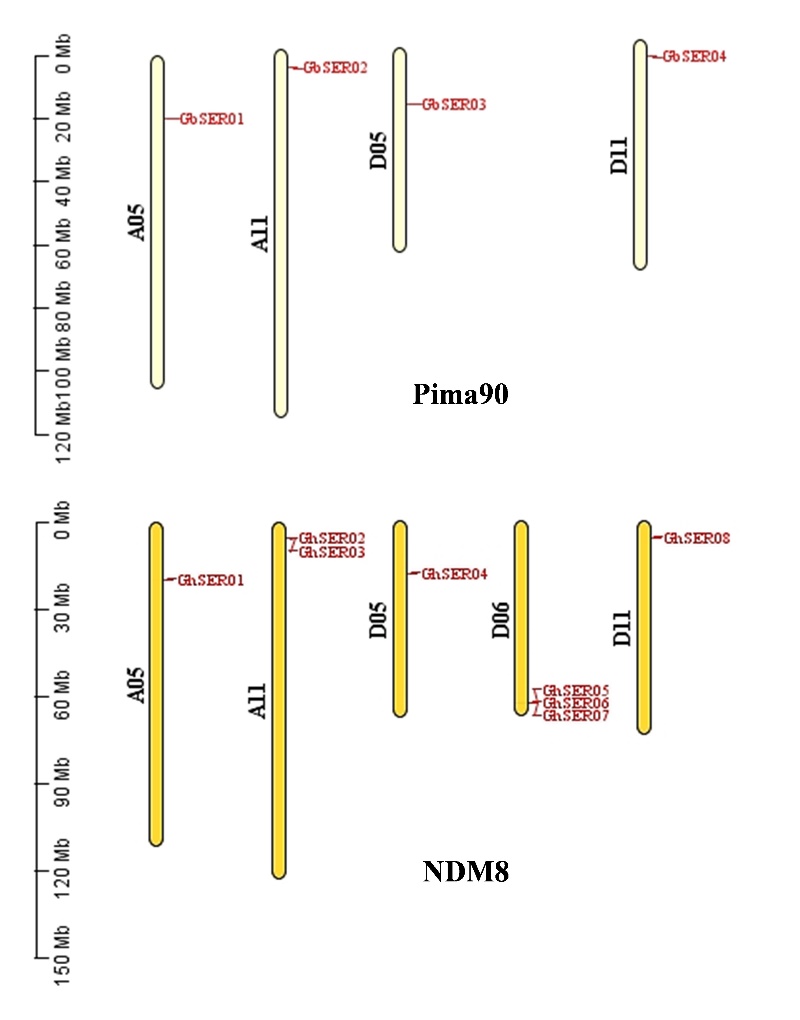


**Supplementary Fig.2 Chromosomal distribution of serpin genes in Pima90 and NDM8.**


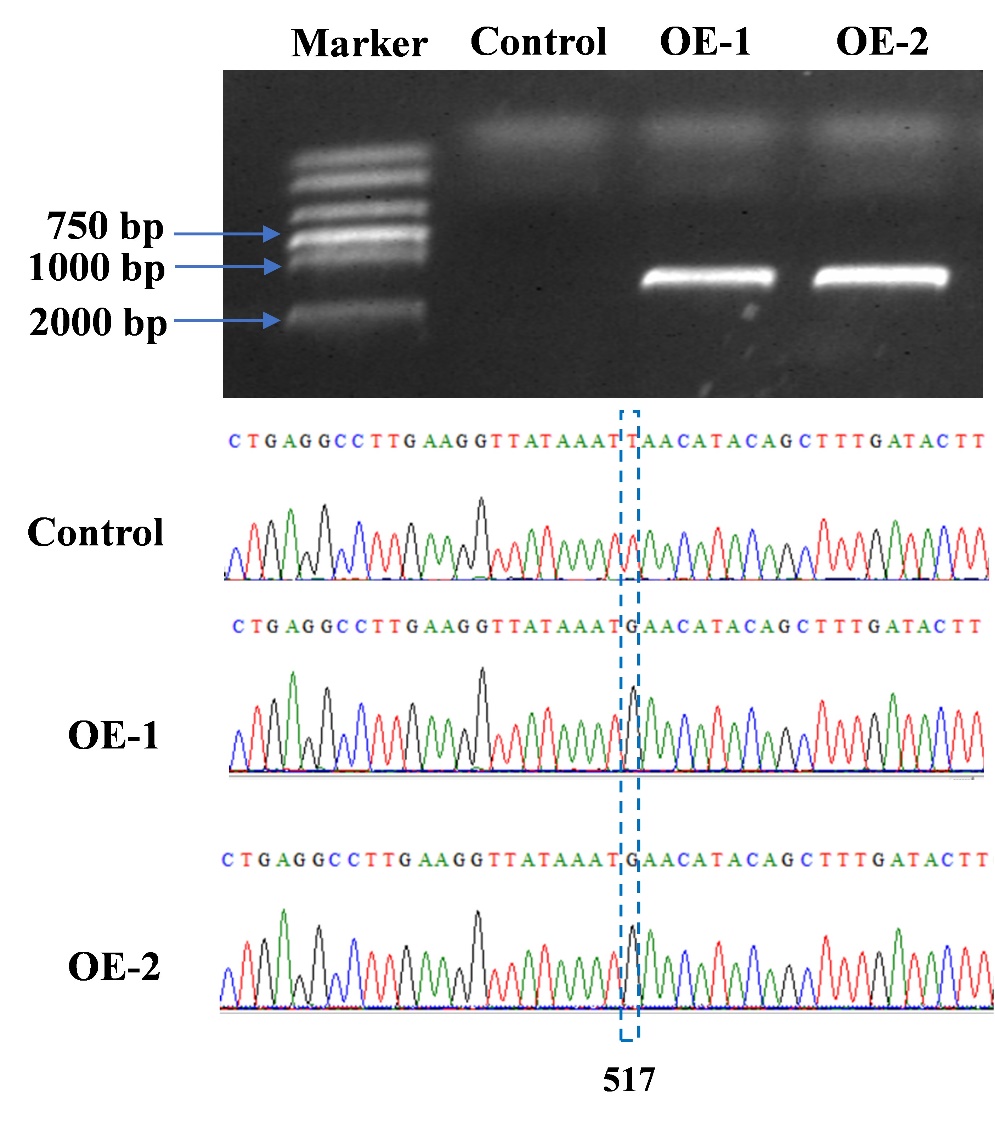


**Supplementary Fig.3 Results of cross-vector primer amplification and product sequencing in transgenic cotton.** The blue dashed box represents important SNP (control is T, both OE-1 and OE-2 are G).


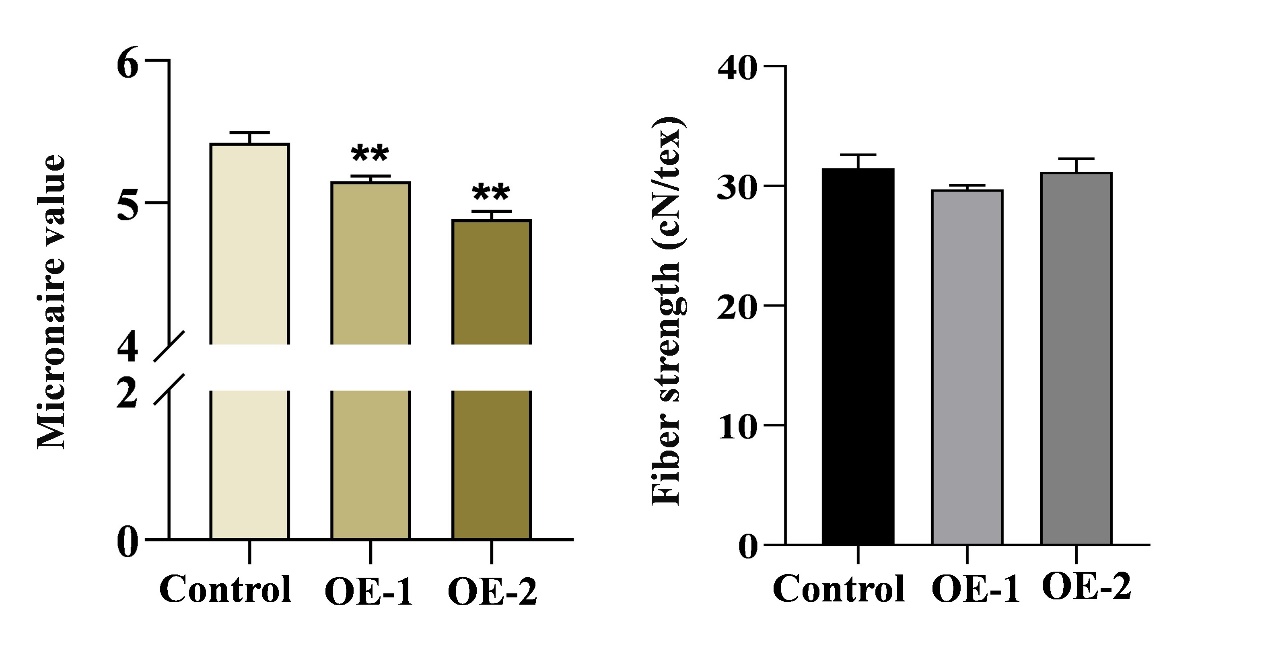


**Supplementary Fig.4 Comparison of Micronaire value and fiber strength between the control and *GbSER02*-OE lines.** ** Significant difference at *P* <0.01 via Student’s *t*-test.


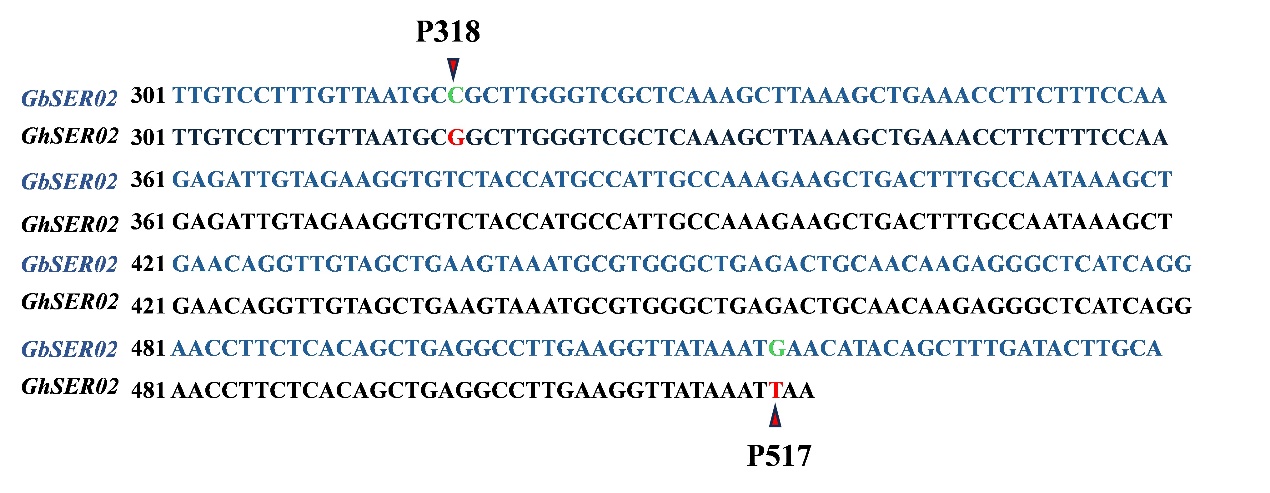


**Supplementary Fig.5 Sequence alignment between *GbSER02* and *GhSER02*.** The arrows indicates the SNPs at positions 318^th^ and 517^th^.


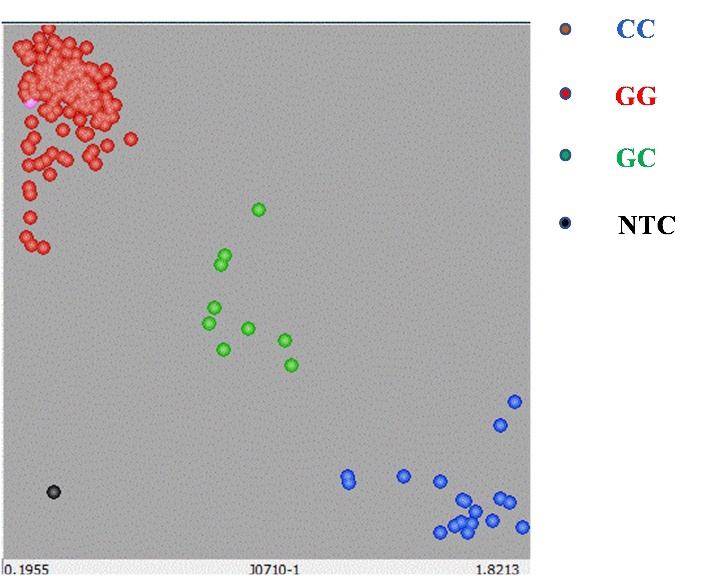


**Supplementary Fig.6 KASP genotyping result.** NTC represents no template control.


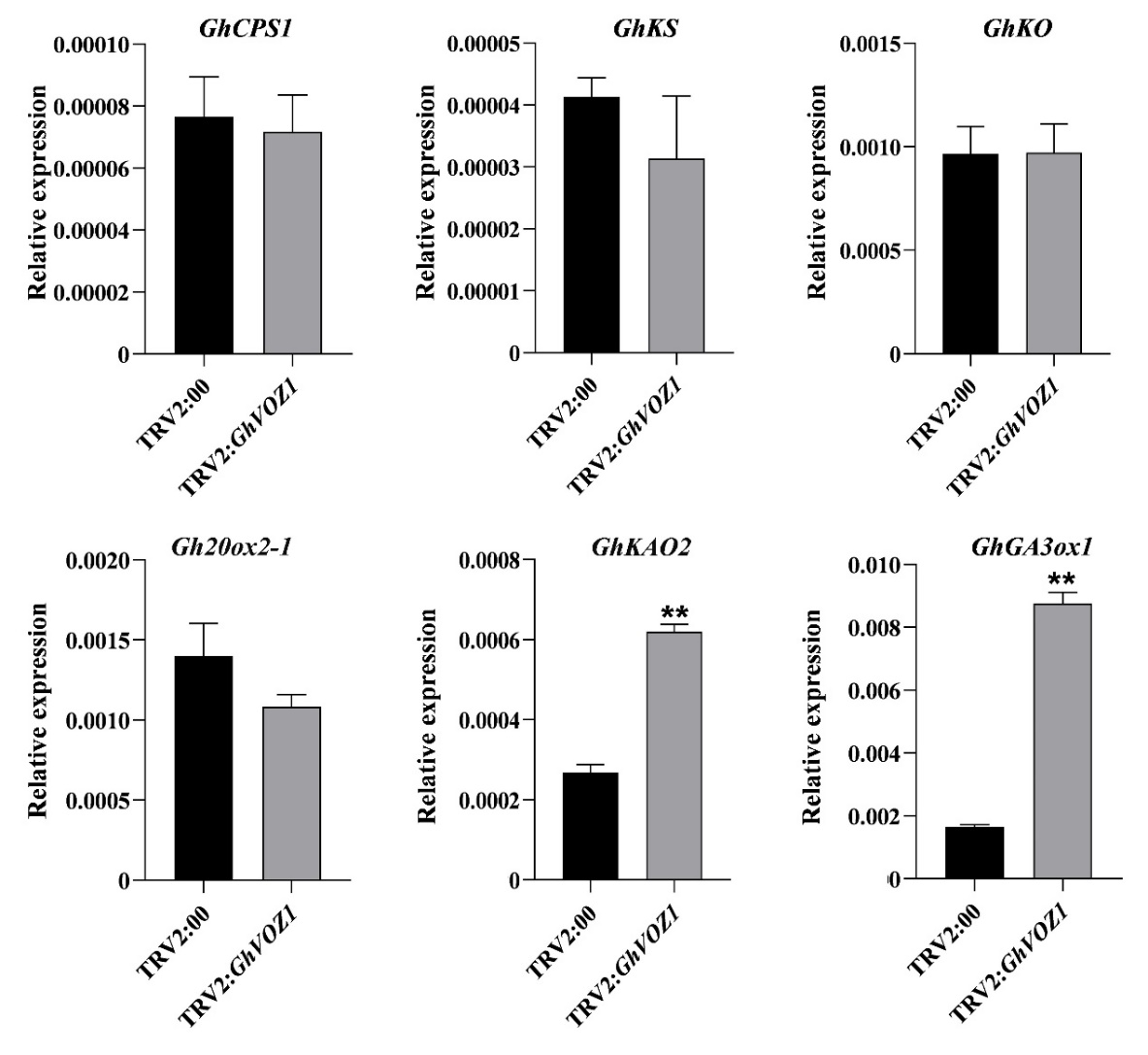


**Supplementary Fig.7 Expression analysis of genes associated with the GA biosynthesis pathway in *GhVOZ1*-silenced plants.** ** Significant difference at *P* <0.01 via Student’s *t*-test.


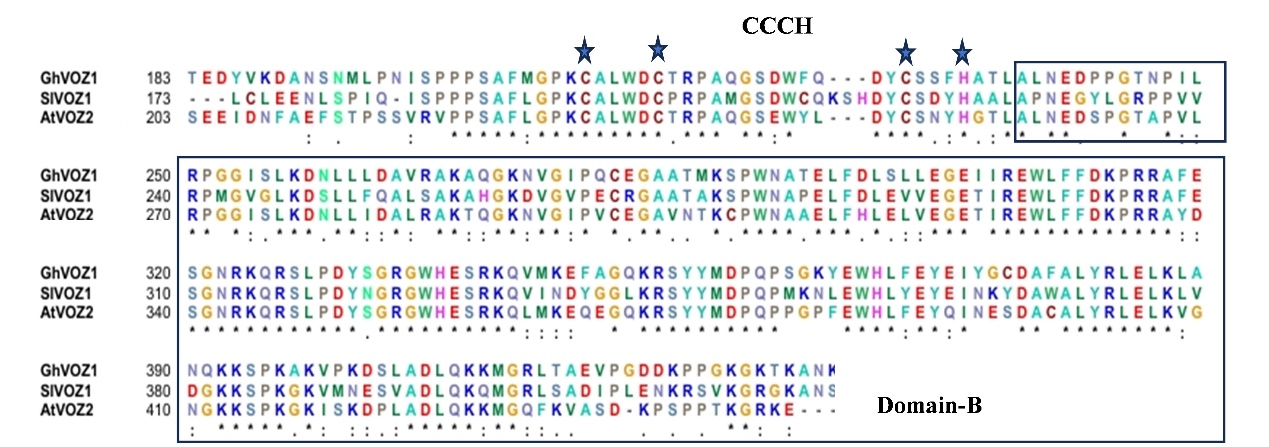


**Supplementary Fig.8 Amino acid alignment of B-domain conserved domains of AtVOZ2 (At2g42400), SlVOZ1, and GhVOZ1.** Asterisks represent conserved amino acid residues that may form a functional zinc-coordinating motif (CCCH), whereas the black box indicates the Domain-B region containing many conserved amino acids.
